# Electrocatalytic Lignin Valorization into Aromatic Products via Oxidative Cleavage of C_**α**_−C_**β**_ Bonds

**DOI:** 10.34133/research.0288

**Published:** 2023-12-15

**Authors:** Jianing Xu, Juan Meng, Yi Hu, Yongzhuang Liu, Yuhan Lou, Wenjing Bai, Shuo Dou, Haipeng Yu, Shuangyin Wang

**Affiliations:** ^1^ Key Laboratory of Bio-based Material Science and Technology of Ministry of Education, Northeast Forestry University, Harbin 150040, China.; ^2^ State Key Laboratory of Chem/Bio-Sensing and Chemometrics, College of Chemistry and Chemical Engineering, Hunan University, Changsha 410082, China.; ^3^ School of Resources and Environmental Engineering, Jiangsu University of Technology, Changzhou 213001, China.

## Abstract

Lignin is the most promising candidate for producing aromatic compounds from biomass. However, the challenge lies in the cleavage of C−C bonds between lignin monomers under mild conditions, as these bonds have high dissociation energy. Electrochemical oxidation, which allows for mild cleavage of C−C bonds, is considered an attractive solution. To achieve low-energy consumption in the valorization of lignin, the use of highly efficient electrocatalysts is essential. In this study, a meticulously designed catalyst consisting of cobalt-doped nickel (oxy)hydroxide on molybdenum disulfide heterojunction was developed. The presence of molybdenum in a high valence state promoted the adsorption of *tert*-butyl hydroperoxide, leading to the formation of critical radical intermediates. In addition, the incorporation of cobalt doping regulated the electronic structure of nickel, resulting in a lower energy barrier. As a result, the heterojunction catalyst demonstrated a selectivity of 85.36% for cleaving the C_α_−C_β_ bond in lignin model compound, achieving a substrate conversion of 93.69% under ambient conditions. In addition, the electrocatalyst depolymerized 49.82 wt% of soluble fractions from organosolv lignin (OL), resulting in a yield of up to 13 wt% of aromatic monomers. Significantly, the effectiveness of the prepared electrocatalyst was also demonstrated using industrial Kraft lignin (KL). Therefore, this research offers a practical approach for implementing electrocatalytic oxidation in lignin refining.

## Introduction

Natural biomass has been emerged as an important candidate to face the anthropogenic climate change and resource depletion crisis caused by the excessive consumption of traditional fossil resources [1–4]. A wide variety of sustainable chemicals, materials, fuels, drug intermediates, etc. could be produced from biomass through facile and mild strategies [5,6]. Lignin as a main component of plant cell walls is the only one with aromatic molecular structures. Hence, it has been placed great expectations in biomass refining to alleviate the dependence on fossil energy [7–9]. This heterogeneous aromatic macromolecule composed of 3 major monolignols, i.e., *p*-coumaryl alcohol, coniferyl alcohol, and sinapyl alcohol, linked by C−C and C−O bonds [10,11]. Their high bond dissociation energy makes lignin highly difficult to chemically process [12,13]. Numerous research has focused on breaking lignin bonds to obtain high-value-added monomers through pyrolysis, hydrogenolysis, and enzymolysis, etc., but conventional approaches require harsh reaction conditions, high energy consumption, costly noble metal catalysts, and are usually associated with suboptimal efficiencies [14,15]. Therefore, a mild and efficient lignin upgrading strategy is urgently needed. Electrocatalytic oxidation is a sustainable depolymerization route for lignin due to its mild reaction conditions at ambient temperature and pressure, easily regulated reaction efficiency, and adjustable product selectivity via accurate potential and current control. It could also offer improved catalyst recyclability by electron transfer between the anode and cathode, which promotes the inner loop redox mechanism of catalytic reaction processes [16–18]. However, because of the complexity of reactant molecular structures and the sluggish electrochemical reaction kinetics, highly efficient and low-cost electrocatalysts are especially needed for selectively depolymerize lignin.

On the other hand, within the lignin, the *β*−O−4 structural units account for over 50% of all linkages, with C_α_−C_β_ bonds being more difficult to cleave than C_β_−O bonds due to their higher bond energy [19–21]. Therefore, directly cleaving C−C bonds presents a pivotal challenge to lignin valorization [22,23]. Previous works have confirmed that the dissociation energy of C_β_−O bonds can be reduced by preoxidizing benzylic alcohols at the C_α_ sites in lignin [24,25]. In contrast, the formation of C_β_ radical intermediates is a key way to achieve C_α_−C_β_ bond cleavage. During this cleavage process, carbon-centered radical intermediates can be formed without substrate pretreatment, allowing lignin depolymerization to be induced in a gentle, clean, and controllable way [26,27]. In a recent study, Pt-based single-atom electrocatalyst was combined with *tert*-butyl hydroperoxide (TBHP) as free radical initiator and radical coupling partner to efficiently cleave the C_α_−C_β_ bonds of lignin [28,29]. The Pt atom anchored C_β_ site in lignin to facilitate the formation of radical intermediates, followed by inducing a radical/radical crossover coupling pathway to weaken the bond dissociation energy, thus overcoming the barrier of C_α_−C_β_ bond cleavage. However, the high cost of noble metal-based materials and the complex catalyst preparation processes also restrict the industrial value-added utilization of lignin. In addition, the favorable adsorption and homolytic cleavage of the free radical initiator TBHP on catalyst surface would be also critical to facilitate the key step of radical intermediates formation during the reaction pathway. Thereinto, transition-metal-based materials such as Ni, Co, Cu, and Mn are widely used in electrocatalysis due to their high activities, abundant reserves, low price, and nontoxicity [30–32]. Ni-based catalysts have demonstrated their feasibility to electrocatalytically depolymerize lignin [33,34]. Simultaneously, as its adjacent element in the periodic table, Co owns similar ionic radius and electronegativity that could lead to better catalyst stability and enhance catalytic performance through precise regulation of electronic surroundings for Ni by doping [35–37].

In this work, we present an electrocatalytic oxidation approach to highly selective cleavage of C_α_−C_β_ bonds of lignin using a Mo@NiCoOOH heterojunction catalyst. To achieve this, 2-dimensional (2D) MoS_2_ was used as a sacrificial template due to the high atomic utilization and easy surface modification. This allowed better graft Ni and Co ions to form NiCo hydroxide on the surface. After electrochemical activation of the NiCo hydroxide–MoS_2_ preelectrocatalyst under oxidation potential, unsplit heterojunction structures were formed with trace amounts of Mo- and Co-doped NiOOH. Mo existed in a high valence state, which promoted the adsorption of TBHP to form critical radicals, i.e., *^t^*BuO**·** and OH**·** under electrocatalysis. The dopant Co atoms regulated the electronic structure of Ni by providing interaction between Co^2+^ and bridging O^2−^ via π-donation [38], weakening the *e*^−^–*e*^−^ repulsion between Ni^2+^ and the O^2−^, eventually promoting partial charge transfer from Ni^2+^ to Co^2+^, and inducing a remarkable increase in Ni^3+^ that acts as the actual catalytic active species. These design principles were dedicated to facilitate the full release of C_β_**·** key intermediates to formed the crucial hydroperoxide intermediate with O_2_ (from air) and radical-coupled intermediate with *^t^*BuO**·** (from TBHP), thereby leading to excellent electrocatalytic activity for C_α_−C_β_ bonds cleavage. The results of electrocatalytic reaction showed a conversion rate of >93% for lignin model compound, 2-phenoxy-1-phenylethanol, with >85% total C_α_−C_β_ bond cleavage to produce benzaldehyde, methyl benzoate, and phenol. In addition, we also demonstrated that the electrocatalytic oxidation strategy with the as-designed catalyst realized the depolymerization of OL and industrial KL with aromatic monomer yields of 13 and 5 wt%, respectively.

## Results

### Synthesis and characterization of catalysts

**Fig. 1.**
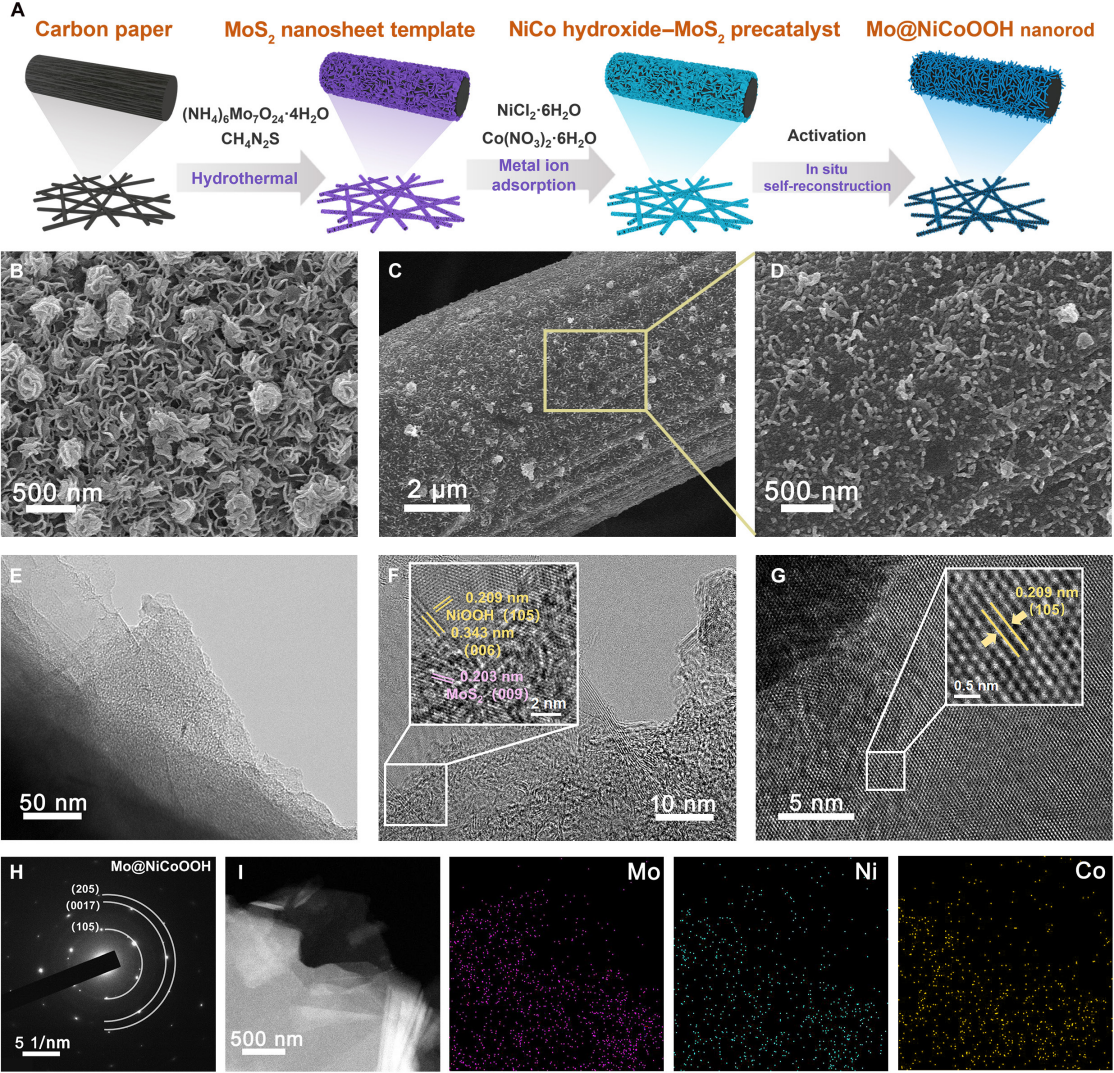
Synthetic strategy and characterizations of catalysts. (A) Schematic illustration of the preparation process of the Mo@NiCoOOH heterojunction. SEM images of (B) the NiCo hydroxide–MoS_2_ precatalyst with nanosheet template and (C and D) Mo@NiCoOOH nanorods at different magnifications. HR-TEM images of (E) low-magnification and (F and G) high-magnification zoom of different regions. (H) SAED pattern and (I) EDS mapping of the Mo@NiCoOOH heterojunction.

The Mo@NiCoOOH heterojunction catalyst was synthesized by an electrochemically mediated in situ self-reconstruction approach based on a MoS_2_ sacrificial template (Fig. 1A). Initially, ultrathin MoS_2_ nanosheets with 1T phase were grown on carbon paper substrates via a hydrothermal reaction (Fig. S1), followed by the physical adsorption of Ni and Co ions from metal precursor solution. The doping ratio of Co could be precisely regulated through this process. NiCo hydroxide–MoS_2_ precatalyst was then obtained after drying (Fig. 1B). Eventually, the cyclic voltammetry activation process was implemented for in situ self-reconstruction by leaching most MoS_2_ template under oxidation condition to obtain the metal (oxy)hydroxide heterogeneous electrocatalysts that retained high-valence Mo.

The morphology of the as-designed electrocatalyst was observed by scanning electron microscopy (SEM) and high-resolution transmission electron microscopy (HR-TEM). The SEM images of the MoS_2_ template with adsorbed metal precursor before and after activation (Fig. 1B to D) show that most of the MoS_2_ nanosheets were removed, and the Mo@NiCoOOH nanorods were created during the reconstruction process. To verify the formation of a heterojunction, HR-TEM was used to investigate the lattice spacing. As shown in Fig. 1E and F, lattice *d-*spacings of 0.209, 0.343, and 0.203 nm were quantified, which corresponded to the (105) and (006) planes of NiOOH and the (009) plane of MoS_2_, respectively. The (105) plane of metal (oxy)hydroxide was the dominant lattice plane in Mo@NiCoOOH (Fig. 1G). Furthermore, the diffraction rings of NiOOH were brighter after Co doping in the selected area electron diffraction (SAED) pattern (Fig. 1H), indicating better crystallinity. The energy-dispersive spectroscopy (EDS) mappings (Fig. 1I) revealed a homogeneous distribution of Mo, Ni, and Co. Comparable physical features could also be observed for Mo@NiOOH (Figs. S2 to S4), validating similar physical properties after Co doping.

**Fig. 2.**
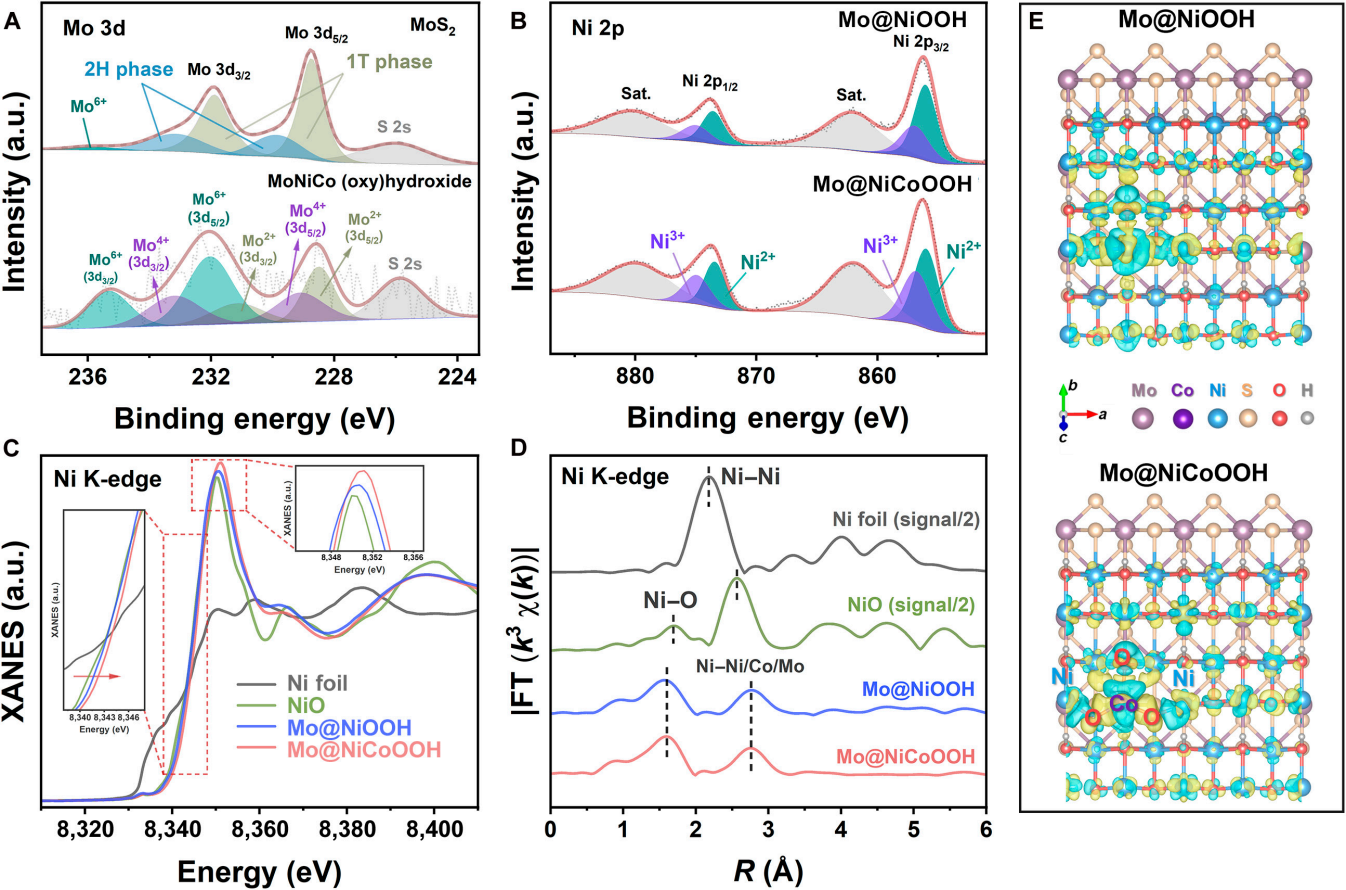
Spectrochemical characterization of catalysts. (A) XPS spectra in the Mo 3d and (B) Ni 2p regions. (C) X-ray absorption near-edge structure (XANES) spectra of the Ni K-edge. (D) Fourier transform (FT) k^3^-weighted EXAFS spectra of Ni K-edge and (E) 3D differential charge densities of Mo@NiOOH and Mo@NiCoOOH optimized structure models. Yellow and blue regions represent excess and depletion of charge density, respectively. a.u., arbitrary units.

X-ray diffraction was conducted to confirm the crystal structures of the catalysts and MoS_2_ template. As seen in Fig. S5, the main diffraction peaks of the as-prepared catalysts located at 43.3° and 44.5°, corresponding to the (105) plane of NiOOH (PDF#06-0075) and (009) plane of MoS_2_ (PDF#17-0744), which was consistent with the TEM results. Other weak diffraction peaks belonging to the (0017) and (205) planes also appeared because of the low loading mass of Mo@NiCoOOH. No extra peaks of other species were observed, highlighting the dominant position of Ni-based catalysts, which was attributed to the leaching of MoS_2_ during electroactivation (Fig. S6). Moreover, to investigate the chemical states of Mo and Ni after Co doping, x-ray photoelectron spectrometry (XPS) was used. In Fig. 2A, the Mo 3d spectrum of Mo@NiCoOOH was split into Mo 3d_3/2_ and 3d_5/2_ peaks at 231.9 and 228.7 eV [39,40]. Following electrochemical activation, the heterojunction showed a considerably higher content of Mo^6+^ than the original MoS_2_ template. It has been demonstrated that the presence of Mo^6+^ species would facilitate the adsorption of TBHP during electrocatalytic depolymerization [41,42]. This phenomenon was also observed for the Mo@NiOOH catalyst without Co doping, implying that the electronic transfer of Mo in the heterojunction was unrelated to Co (Fig. S7). The Ni 2p spectra (Fig. 2B) showed 2 significant peaks at 873.7 and 856.2 eV, which corresponded to the characteristic binding energies of Ni 2p_1/2_ and 2p_3/2_. The deconvoluted peaks with binding energies at 874.9 and 856.8 eV agreed with Ni^3+^, whereas the peaks centered at 873.5 and 856.0 eV corresponded to Ni^2+^ [43,44]. Because of the doping of Co atoms, the electronic structure of Ni atoms may have changed, which greatly increased the ratio of Ni^3+^ to Ni^2+^. As demonstrated that the Ni^3+^ is the actual catalytic active site for lignin electrooxidation, the introduction of Co is vital for boosting the catalytic activity of NiOOH. In addition, the XPS spectrum of Co was deconvoluted into peaks of Co^2+^ and Co^3+^ (Fig. S8) [45]. The actual loading amounts of Ni, Co, and Mo were determined using inductively coupled plasma-optical emission spectrometry as 30.01, 6.29, and 0.70 μg/cm^2^ in the Mo@NiCoOOH electrode, respectively (Fig. S9).

X-ray absorption fine structure (XAFS) measurements were performed to further investigate the chemical states and coordination environments of Ni atoms in Mo@NiOOH and Mo@NiCoOOH. The oxidation states of Ni atoms in the samples at the K-edge in the x-ray absorption near-edge structure spectra are shown in Fig. 2C (with Ni foil and NiO as references). As can be seen in the partially enlarged details from 8,338 to 8,348 eV, the K-edge curves of both Mo@NiOOH and Mo@NiCoOOH were located at higher energies than NiO, revealing the formation of Ni^3+^ active species by our electrochemical mediated in situ activation strategy. The absorption edge of Ni in Mo@NiCoOOH showed a more pronounced right shift to Mo@NiOOH, implying significant electron loss of Ni after Co doping. This conclusion was also supported by the absorption peaks in the magnified area of ca. 8,344 to 8,357 eV, where the peak intensity was much stronger than that of Mo@NiOOH and NiO reference samples [46].

The coordination configurations were further examined by Fourier transform extended XAFS (EXAFS) spectra in the *R*-space (Fig. 2D). It was observed that the EXAFS spectrum of Mo@NiCoOOH presented a similar profile to that of Mo@NiOOH without Co doping, indicating that the local Ni coordination environment was similar in both samples. Thus, the increased oxidation state of Ni caused by Co doping was not due to a change in the coordination environment. Note that the distinctly shorter Ni−O scatterings (near 1.6 Å) of Mo@NiCoOOH and Mo@NiOOH compared with NiO (locate around 1.7 Å) caused stronger Ni−O bonds [47]. For the second and higher-shell structures (*R* > 2 Å), which represent Ni−Ni/Co/Mo interactions, the peaks located around 2.75 Å in the 2 prepared catalysts were observably different from that of NiO (2.57 Å) but well matched to NiOOH (2.82 Å) [48,49]. The synchrotron x-ray absorption spectroscopy (XAS) characterization analysis strongly supports our hypothesis of the successfully formed Mo@NiOOH heterostructure, while Co doping afforded Ni greater electron loss and richer Ni^3+^ active species. To gain further insight into the electron transfer in catalysts, the optimized theoretical models of Mo@NiOOH and Mo@NiCoOOH were established by theoretical calculations as shown in Fig. S10. Ni and Co, as transition metals, have strong electron correlation, which could provide a more available and broader charge rearrangement for Ni-based materials by Co doping. Thus, the distribution of active sites, conductivity, and catalytic performance would change accordingly. The charge redistribution can be imaged by 3D differential charge densities in Fig. 2E. A distinct excess of charge density (yellow) around O and Co atoms could be observed in the doping area of Mo@NiCoOOH compared to Mo@NiOOH. In addition, the adjacent Ni atoms showed a depletion of charge density (blue) after Co doping, while Mo atoms were barely affected, which is consistent with the XPS results. This phenomenon can be attributed to the interaction of introduced Co^2+^ and bridging O^2−^ via π-donation, weakening the *e*^−^–*e*^−^ repulsion between Ni^2+^ and bridging O^2−^, eventually promoting partial charge transfer from Ni^2+^ to Co^2+^ (Fig. S11) [50]. The charge rearrangement will provide Mo@NiCoOOH with well-matched adsorption/activation sites for activating or forming certain chemical bonds to achieve improved catalytic activities.

### Electrocatalytic oxidation of 2-phenoxy-1-phenylethanol

**Fig. 3.**
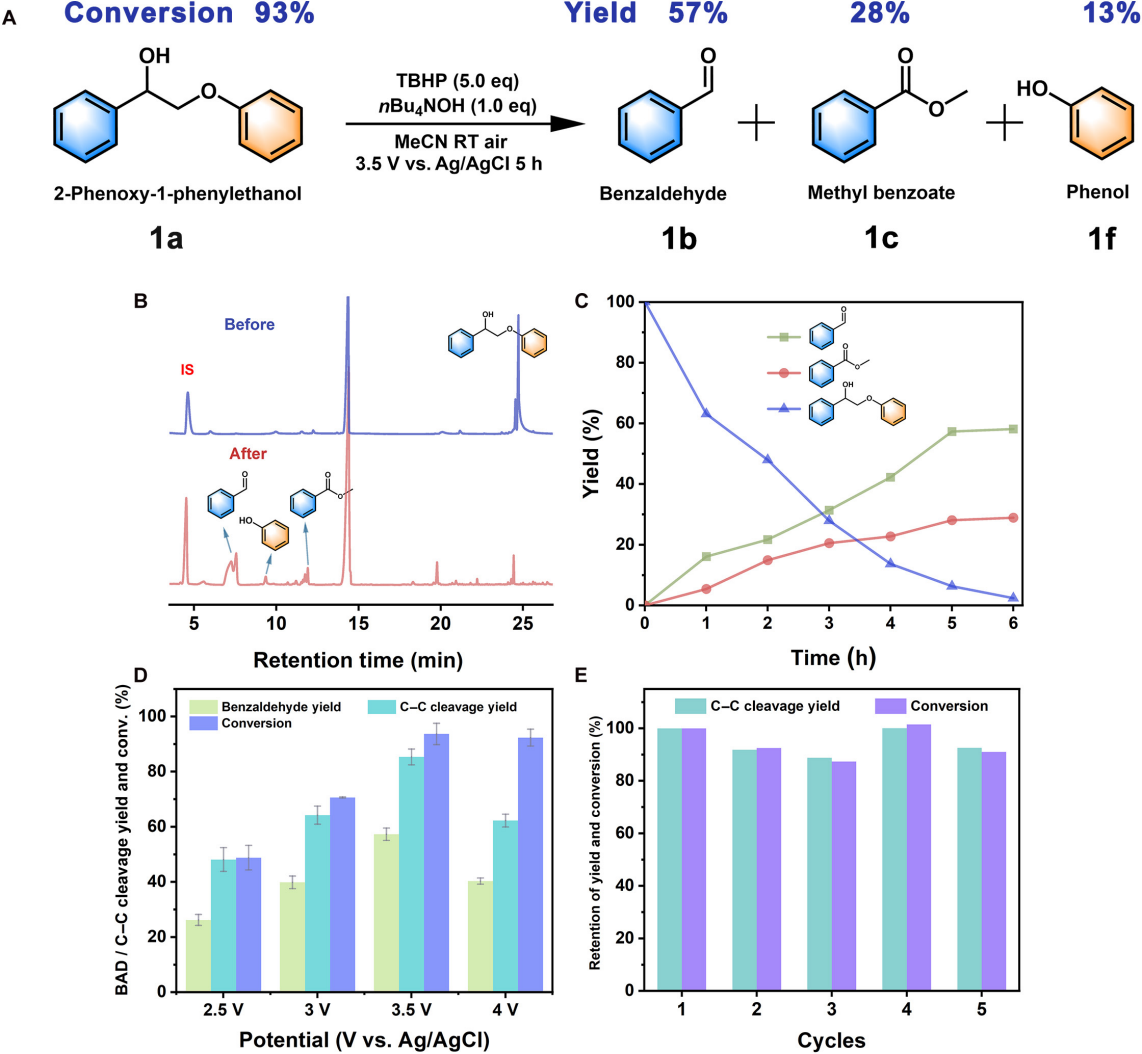
Catalytic conversion of lignin model compound (2-phenoxy-1-phenylethanol). (A) Schematic of the oxidative cleavage reaction. Standard reaction conditions: 1a (0.2 mmol), *n*Bu_4_NOH (0.2 mmol), TBHP (1.0 mmol), MeCN (10.0 ml), internal standard (IS), RT (room temperature), potential = 3.5 V versus Ag/AgCl, 5 h, under air. (B) GC chromatograms before and after the electrocatalytic oxidative reaction. (C) Overall time course of the reaction of the substrate (1a) and products (1b, 1c, and 1d) under standard reaction conditions. (D) The yield of main product 1b (BAD, benzaldehyde) C−C bond cleavage, and the conversion of 1a under different potentials. (E) Cycling evaluations on the reusability of Mo@NiCoOOH.

The most representative dimeric lignin model compound, 2-phenoxy-1-phenylethanol (**1a**), was selected as the substrate to investigate the C_α_−C_β_ bond cleavage regularity and establish the optimal reaction conditions under the catalysis by the Mo@NiCoOOH heterojunction. Electrocatalytic oxidation reactions were carried out in an undivided cell with a standard 3-electron system equipped with Mo@NiCoOOH as the working electrode, platinum (Pt) as the counter electrode, and Ag/AgCl as the reference electrode. As stated, the cleavage of C_α_−C_β_ bond relied primarily on the formation of C_β_**·** intermediate at catalytic active sites and bonding with O_2_ or *^t^*BuO**·** radicals to lower the bond dissociation energy. Thus, TBHP (55% aqueous solution) was used as the radical initiator and coupling partner in this work. As illustrated in Fig. 3A, the in situ formed *^t^*BuO**·** derived from the homolytic cleavage of TBHP was the main radical that participate in crucial radical intermediates formations with the C_β_**·** intermediate from **1a** to realize the electrocatalytic oxidative cleavage of C_α_−C_β_ bonds. The catalytic activities and the bond cleavage selectivity were studied through qualitative and quantitative analyses by gas chromatography-mass spectrometry (GC-MS) and GC. Figure 3B shows that under constant electrocatalytic conditions, the main products of **1a** were benzaldehyde (**1b**), methyl benzoate (**1c**), and phenol (**1f**) by the distinctly desired C_α_−C_β_ bonds cleavage, without other obvious unrelated monomer by-products produced and an almost complete conversion of **1a** was achieved.

To reveal the role of Co doping, Mo@NiCoOOH with varying amounts of Co was synthesized and denoted as Mo@Ni_10−*x*_Co*_x_*OOH (*x* = 0, 1, 2, 3, 10). As seen in Fig. S12, the electrocatalytic depolymerization activities of Mo@Ni_10−*x*_Co*_x_*OOH samples displayed a strong dependence on the Co content. Mo@NiOOH (*x* = 0) showed a C_α_−C_β_ bond cleavage yield and **1a** conversion rate of 76.54% and 92.96%, respectively, with selectivity for C_α_−C_β_ bond cleavage at 82.69%. When 10% Co was introduced to the heterojunction (Mo@Ni_9_Co_1_OOH, *x* = 1), we obtained a C_α_−C_β_ bond cleavage products yield of 85.36% including **1b** and **1c** for 57.29% and 28.07%, meanwhile a yield of 13% for phenol. The conversion rate of **1a** reached 93.69%, and the selectivity of C_α_−C_β_ bond cleavage achieved 91.11% as the optimal result. However, further increasing the proportion of Co led to inferior yields and conversion rates. This owns to that more catalytic active sites (Ni atoms) were substituted with Co atoms, suppressing their interactions with reactants. To verify the synergistic catalysis of the heterojunction on the MoS_2_ substrate, pure Ni_9_Co_1_OOH and MoS_2_ were directly hydrothermally synthesized on carbon paper and subjected to the same electrooxidation reactions, but low conversion rates of **1a** and poor product selectivity were obtained (Figs. S13 and S14). These comparisons highlight the important role of high-valence Mo and heterostructure in the electrocatalytic activity of the Mo@NiCoOOH catalyst, with the Mo@Ni_9_Co_1_OOH exhibiting the highest electrocatalytic activity among all samples. Thus, all other experiments were carried out on the basis of the Mo@Ni_9_Co_1_OOH, directly named as Mo@NiCoOOH unless otherwise state.

Time-gradient electrolysis experiments (from 0 to 6 h) were conducted to explore the optimal reaction times. As can be seen in Fig. 3C, when prolonging the electrolysis time, substrate **1a** was gradually consumed, and the main products **1b** and **1c** were observed with satisfactory product specificity maintained under the standard conditions, suggesting a highly selective C_α_−C_β_ bond cleavage by Mo@NiCoOOH. Depolymerization was almost complete at 5 h, and the yields barely increased when the reaction time was extended to 6 h. To study the effect of potentials on the bond cleavage activity, electrocatalysis was further performed at different potentials using Mo@NiCoOOH (Fig. 3D). The GC quantification (Fig. S15) suggested that 3.5 V was the optimal potential for electrocatalytic oxidative cleavage with a current density of ~13 mA/cm^2^ (Fig. S16). Lower potential did not achieve sufficient catalytic efficiency, while higher potential had difficulty in maintaining product selectivity due to poorly matched electron transfer between the electrode and reaction intermediates. Another important criterion for an electrocatalyst is its operational stability. Therefore, cyclic electrolysis experiments were performed with reuse cycles. The yields and conversion rates did not decrease significantly (Fig. 3E) and always maintained over 85% of the original catalytic efficiency in the first 5 cycles, showing an excellent stability of the Mo@NiCoOOH heterojunction catalyst. The conversion capacity of catalyst had a relatively decline until the 10th cycle (Fig. S17), and this phenomenon may be put down to a densified surface reconfiguration of the catalyst after prolonged use (Fig. S18). Then, the electrocatalytic performance of the noble metal-free Mo@NiCoOOH heterojunction was compared with recently reported catalysts for electro-, photo-, and thermal approaches (Table S1). We also choose the 2-(2-methoxyphenoxy)-1-phenylethanol, which more closely resembles the native lignin structure as other substrate, to verified the universal C_α_−C_β_ bond cleavage ability in Fig. S19. As a result, the products **1b, 1c**, and guaiacol were detected with yields of 32%, 15% and 9%, respectively. The competitive catalytic efficiency of the substrate dosage, short-time consumption, and convenient experimental conditions provide a facile strategy for the electrocatalytic depolymerization of lignin.

### Mechanism of electrocatalytic C**_α_**−C_β_ bond cleavage by Mo@NiCoOOH

To gain a better understanding of the mechanism for electrocatalytic C_α_−C_β_ bond oxidative cleavage, serial control experiments were carried out along with density functional theory (DFT) calculations. First, the radical reaction pathway was studied by adding the radical scavenger, 2,2,6,6-tetramethyl-1-piperidinyloxy (TEMPO) [51]. As seen in Fig. S20A, the GCs of reaction mixtures before and after electrocatalytic oxidative reaction with TEMPO showed no differences in terms of substrate consumption and product formation. This suggests that the cleavage of C_α_−C_β_ bonds was prevented by inhibiting the formation of key radical intermediates.

**Fig. 4.**
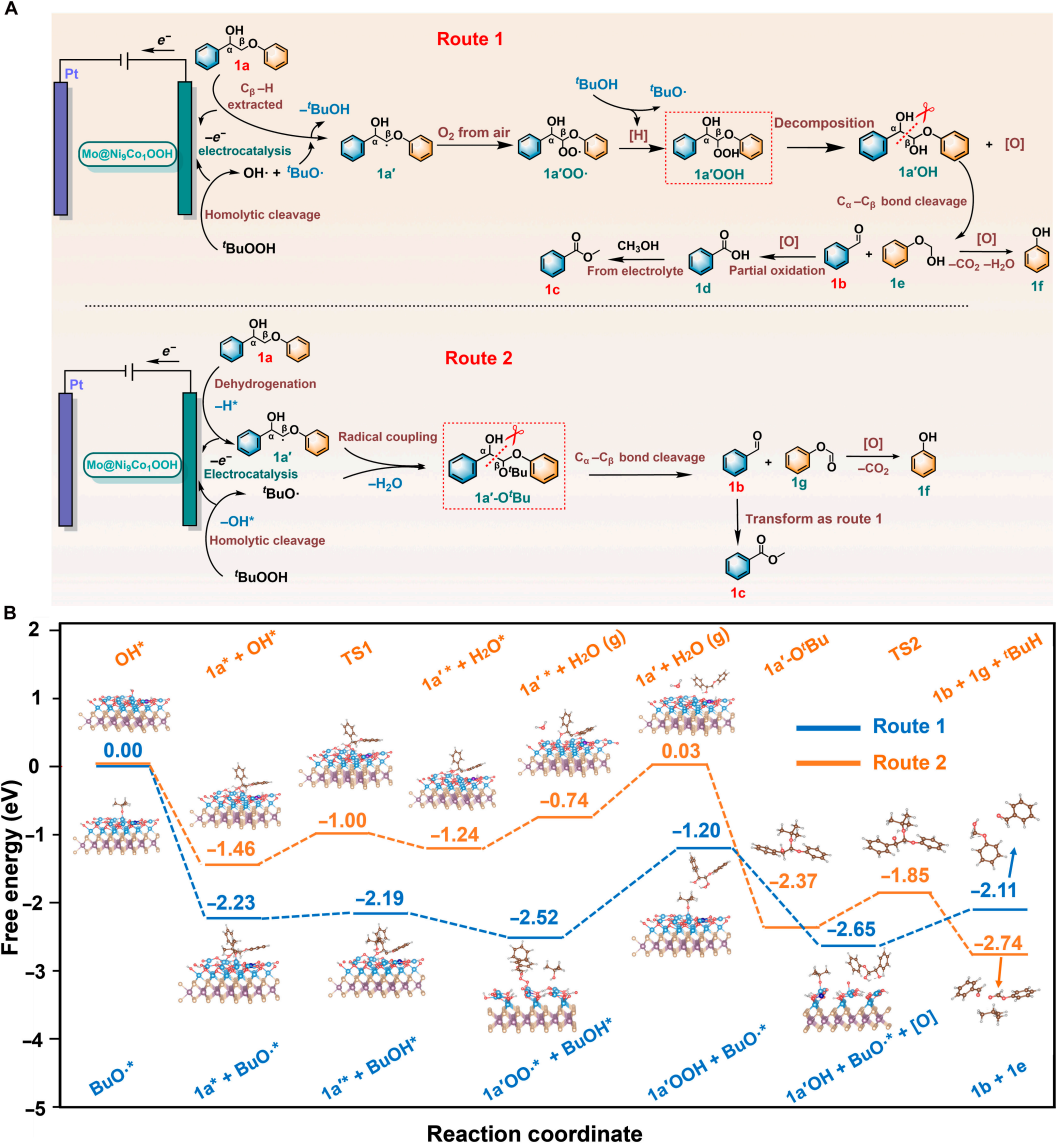
Reaction pathways based on experiments and DFT calculations. (A) Proposed mechanisms of Mo@NiCoOOH-catalyzed conversion of 1a. (B) The DFT-calculated Gibbs free energy diagram and optimized reaction intermediate structures for 1a conversion on the Ni active sites of Mo@NiCoOOH for 2 routes.

The proposed C_α_−C_β_ bond oxidative cleavage pathways for **1a** over the Mo@NiCoOOH catalyst are shown in Fig. 4A. As reported in previous literature, TBHP could play important roles as radical initiator, terminal oxidant, and radical coupling partner in the reaction by decomposing into *^t^*BuO**·** and OH**·** [52]. We experimentally verified that the C_α_−C_β_ bond cleavage goes through 2 pathways at once by forming different key intermediates. For the route 1, O_2_ from air participated in the formation of **1a′OO·** with **1a′** which extracted C_β_–H from **1a** by *^t^*BuO**·** [53–58]. Next, the intermediate **1a′OO·** bind with the H of *^t^*BuOH to form **1a′OOH** and then decomposed into **1a′OH** and **[O]**. Finally, benzaldehyde (**1b**) and **1e** were formed through the C_α_−C_β_ bond cleavage under the promotion of electrocatalysis. Part of the **1b** was further oxidized into benzoic acid (**1d**) and then esterized with methanol from electrolyte to produce methyl benzoate (**1c**). Meanwhile, **1e** transformed into phenol (**1f**) by dehydration and CO_2_ removal. This route could afford part of products, and the remaining was produced through radical coupling in route 2 that C_β_–H was extracted by electrocatalytic oxidation in anode [59,60]. This was confirmed by experiments under the N_2_ atmosphere as shown in Fig. S20B, since the products could be detected without O_2_. In this pathway, C_β_**·** radical **1a′** was generated via C_β_−H transfer from **1a** to the present OH^*^ at Ni site (adjacent to Co) of Mo@NiCoOOH. Subsequently, the unstable radical intermediate *^t^*BuO**·** and **1a′** underwent radical/radical cross-coupling to form **1a′-O**^*t*^**Bu**. Finally, electron transfer in **1a′-O**^*t*^**Bu** led to the selective cleavage of C_α_−C_β_ bonds, producing **1b** and **1g**. In the reaction mixture, **1c** was formed as route 1, and the unstable intermediate **1g** spontaneously decomposed into **1f** and CO_2_. The reason for the relatively low retention rate of phenol is attributed to the hydroxyl radicals from *^t^*BuOOH in the electrocatalytic reaction mixture, one of the strongest inorganic oxidants, oxidized part of the highly degradable compound **1f** into free radicals. Further chain oxidation of the organic radicals led to a decomposition of **1f** into CO_2_ and water facilitated by abundant electron transfer in electrocatalysis [61,62]. A control experiment, in which substrate **1a** was replaced by phenol, was carried out to prove this conclusion. The GC result (Fig. S21) showed an obvious degradation of phenol after a long-term electrocatalytic oxidation process, which corroborated the radical reaction pathway.

DFT calculations were further performed in Fig. 4B to investigate the reaction energy profiles of these 2 routes by establishing optimized theoretical models. As reported by previous work from Duan’s group [28], C_β_–H of **1a** was the decisively adsorbed site, and the *^t^*BuO**·** was more resultful to participate in the radical-mediated cleavage reaction than *^t^*BuOO**·**. We can find out that the rate-determining step (RDS) of route 1 was the formation of **1a′OOH** intermediate by hydrogen transfer from *^t^*BuOH, with a Gibbs free-energy change (Δ*G*) of 1.32 eV, which is 0.55 eV higher than the RDS in route 2 (**1a′** desorption, Δ*G* = 0.77 eV). Nevertheless, the overall Gibbs free energy is lower than that of route 2. This phenomenon confirmed that the formation of **1a′OOH** hydroperoxide intermediate is much crucial for promoting the formation of products, which corresponds well with the experimental results. Under the promotion of electrocatalysis, the 2 pathways work together to bring out the excellent substrate conversion.

Moreover, to confirming the superiority of Co doping, we also explored the theoretical adsorption and reaction behaviors on Mo@NiOOH and Mo@NiCoOOH. Taking route 2 as example (Fig. S22), the calculated adsorption energies (*E*_ads_) of **1a** on Mo@NiOOH and Mo@NiCoOOH were −1.03 and −1.50 eV, respectively. This demonstrated that Co doping provided Ni active site with a more accessible substrate adsorption ability. More importantly, the energy diagram (Fig. S23) shows that the RDS (**1a′** desorption) of Mo@NiCoOOH with a Δ*G* of 0.77 eV was much lower than Mo@NiOOH (2.41 eV). All the results signify that Co doping effectively reduces the required energy for C_α_−C_β_ bond cleavage process and strongly improves the catalytic performance of Mo@NiCoOOH catalyst (Tables S2 and S3).

### Practical application toward the electrocatalytic oxidation of lignin

**Fig. 5.**
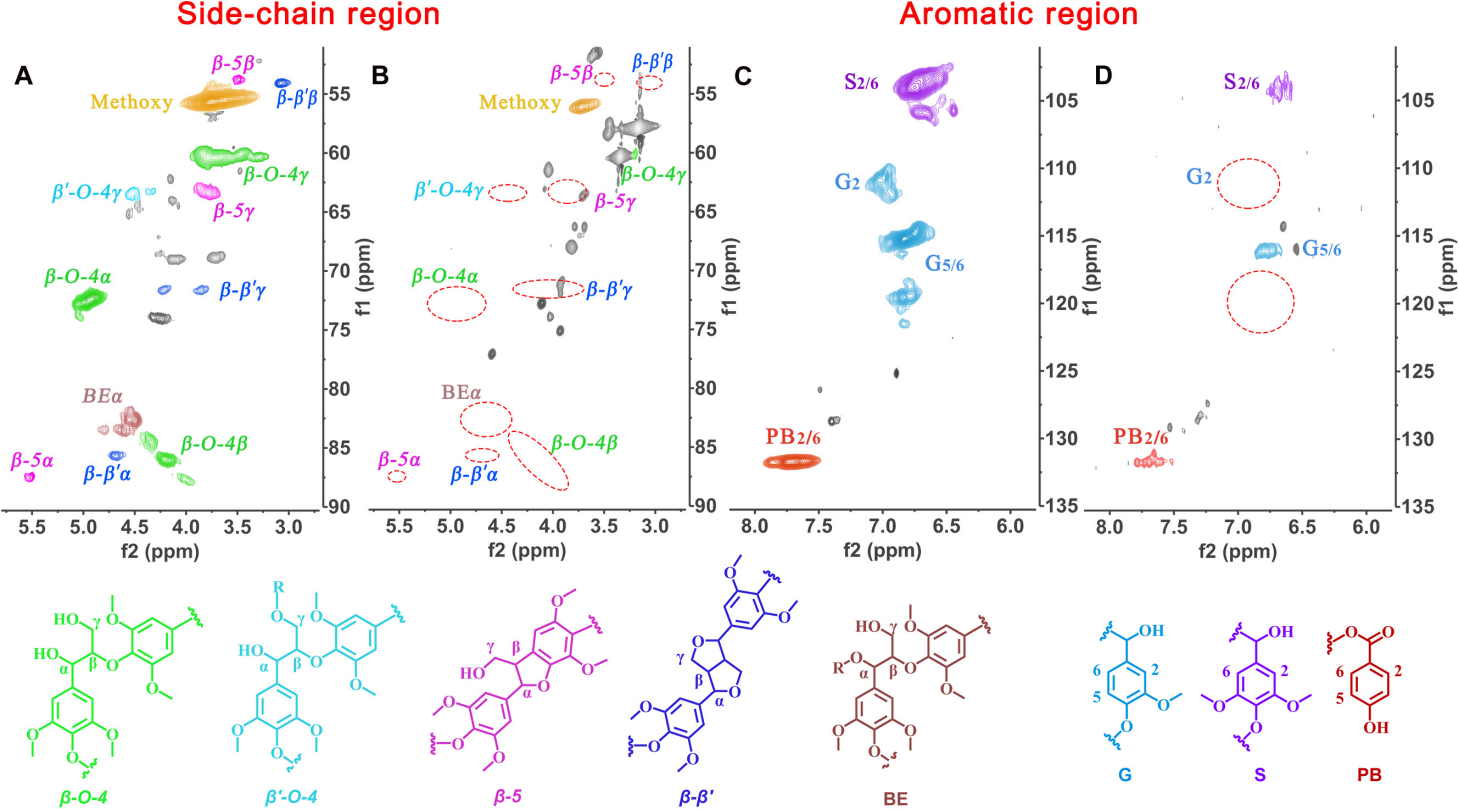
2D HSQC NMR spectra of OL. (A) Before and (B) after the electrocatalytic reactions in side-chain regions. (C) Before and (D) after the electrocatalytic reactions in aromatic regions. Reaction conditions: OL (50 mg), *n*Bu_4_NOH (0.22 mmol), TBHP (1.2 mmol), 2-methyltetrahydrofuran (2-Me THF) (5.0 ml), MeCN (5.0 ml), RT, potential = 4.0 V versus Ag/AgCl, 10 h, under air.

Inspired by the excellent electrocatalytic activity and C_α_−C_β_ bond cleavage selectivity, we explored the depolymerization of native lignin under electrocatalysis by Mo@NiCoOOH. OL extracted from poplar via acid treatment in dioxane was used as the substrate, and the electrocatalytic process was set to a slightly higher potential (4 V versus Ag/AgCl; Fig. S24) to cope with the more complex macromolecular structures compared with the dimeric model compound. 2D heteronuclear single quantum coherence nuclear magnetic resonance (2D HSQC NMR) spectra were obtained to detect the OL structure before and after electrocatalytic reactions of the main bonds (Fig. 5). In the side-chain region of OL (Fig. 5A), typical signals of *β*-O-4, *β*-5, and *β*-*β* linkages were observed [63,64]. After the electrocatalytic reaction under ambient conditions, signals belonging to the C_α_−C_β_ bonds (*α* and *β* sites of *β*-O-4, *β'*-O-4, *β*-5, *β*-*β'*, and benzyl ether) disappeared (Fig. 5B), and the *γ* sites’ signals were also significantly weakened. This demonstrates that the C_α_−C_β_ bonds were destroyed as per our expectations. The decrease in the aromatic region of guaiacyl (G), syringyl (S), and *p*-hydroxybenzoate units after depolymerization was also observed, as shown in Fig. 5C and D, further confirming the production of lignin monomers [65].

**Fig. 6.**
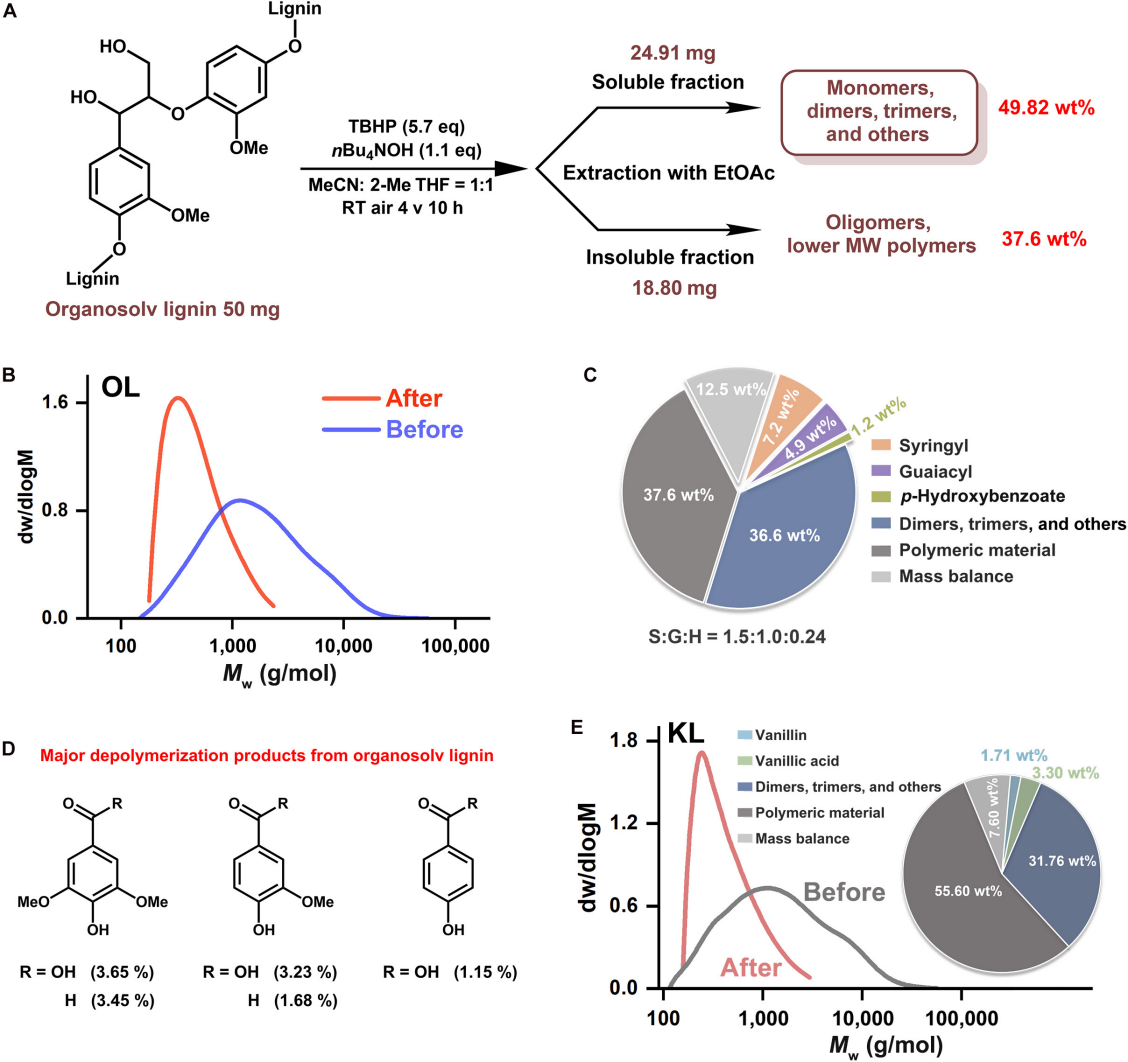
Depolymerization of OL with Mo@NiCoOOH. (A) Schematic of the whole process for the electrocatalytic valorization of OL. (B) GPC of the residue mixture fraction after (red trace; *M*_w_ = 568 g/mol) reaction and original OL (blue trace, *M*_w_ = 2651 g/mol). (C) Identification, quantification, and distribution of the reaction mixture obtained from the oxidative electrocatalytic depolymerization of OL. (D) The major aromatic monomer products from OL. (E) GPC comparison of the residue mixture fraction after (pink trace; *M*_w_ = 557 g/mol) reaction and original KL (gray trace; *M*_w_ = 2768 g/mol), including identification, quantification, and distribution of the reaction mixture obtained from the oxidative electrocatalytic depolymerization of KL.

We conducted qualitatively and quantitatively analysis on the collected products. The solvent in the mixture solution after the reaction was removed by rotary evaporation, and the residue was extracted with ethyl acetate. A soluble fraction was obtained, which accounted for 49.82 wt% of the original lignin, together with an insoluble fraction that accounted for 37.6 wt% (Fig. 6A). The reaction residue was analyzed by gel permeation chromatography (GPC), and the results revealed a significant decrease in molecular weight compared with the OL (Fig. 6B). GC-MS results of the soluble fraction revealed that OL was depolymerized into aromatic monomers, mainly including vanillin and syringaldehyde along with some dimers and trimers (Figs. S25 to S30). From the quantitative GC results, over 13 wt% of the OL was converted to oxidized aromatic compounds through electrocatalytic depolymerization by Mo@NiCoOOH (Fig. 6C). Aromatic acids and aldehydes derived from S, G, and *p*-hydroxybenzoate were the major depolymerization products (7.2, 4.9, and 1.2 wt%), which consisted of the functional groups in the lignin model compounds. The S:G product ratio (1.5:1) was not very different from the S:G monomer ratio in original lignin (Fig. 6D) [66].

To further expand the applications of the electrocatalyst on various lignin, industrial Kraft lignin was also studied as a reactant under the electrocatalysis of the as-prepared Mo@NiCoOOH. From the aromatic region results in the 2D HSQC NMR spectrum (Fig. S31), it can be determined that the used Kraft lignin originated from softwood and the corresponding aromatic monomer products (vanillin and vanillic acid) (Fig. S32) were obtained through continuous electrocatalysis. The yields of G-derived products, with high commercial value, reached more than 5.0 wt% of Kraft lignin, according to GC analysis (Fig. 6E). All these results confirm that the electrocatalytic system dominated by the Mo@NiCoOOH heterojunction played a significant role in the universal C_α_−C_β_ bond dissociation of various lignin samples.

## Discussion

In this study, we demonstrated that the Mo@NiCoOOH heterojunction can selectively catalyze the cleavage of C_α_−C_β_ bond in both lignin model compounds and native/industrial lignin under ambient conditions via electrocatalytic oxidation. Detailed mechanistic studies based on model compound experiments and DFT theoretical calculations indicated that Mo@NiCoOOH had an excellent catalytic performance for C_α_−C_β_ bond cleavage through the formation of both **1a′OOH** and **1a′-O**^*t*^**Bu** as the key intermediates. Vanillin and other aromatic monomers were obtained in decent yields from native/industrial lignin under ambient conditions. This research verified the preparation of renewable aromatics via a mild electrocatalytic conversion route without using noble metals or harsh reaction conditions. It also marks a significant milestone in the utilization of lignin, as providing a promising avenue to produce valuable chemical feedstock and offering preliminary guidance for the development of lignin electrocatalytic depolymerization.

## Materials and Methods

### Methods

#### Synthesis of Mo@Ni_9_Co_1_OOH [MoNiCo (oxy)hydroxides]

The MoS_2_/NiCo lactate dehydrogenase precatalysts were constructed by a chemical bath. MoS_2_ nanosheets were activated at −1.5 V for 10 min (versus Ag/AgCl) in 1 M KOH to improve the hydrophilia and then washed with deionized water thoroughly. The resulting samples were further immersed in a mixed solution of NiCl_2_·6H_2_O and Co(NO_3_)_2_·6H_2_O (30 mM) for 20 min for physically adsorbing Ni and Co ions on the surface to construct the precatalysts. The doping ratio of 2 metals can be precisely regulated in this process. Then, the precatalysts were washed with deionized water thoroughly to remove excess adsorbate and dried at ambient conditions. After that, the MoS_2_/NiCo lactate dehydrogenase precatalysts were subjected to 5 cycles of cyclic voltammetry activation [the potential range from 0 to 0.8 V (versus Ag/AgCl) with a scan rate of 5 mV/s] in 1 M KOH solution to obtain self-reconstruction Mo doping MoNiCo (oxy)hydroxide through Mo leaching. Finally, the catalyst was successfully prepared by activation using constant potential of 0.7 V (versus Ag/AgCl).

#### Electrocatalytic depolymerization of lignin model compound

Electrochemical measurements were performed using a 3-electrode system in a CHI-660E electrochemical station. MeCN was used as the solvent with the addition of *n*Bu_4_NOH (1.0 M MeOH solution) to constitute the organic electrolyte. Mo@Ni_9_Co_1_OOH was used as the working electrode with a Pt counter electrode and Ag/AgCl reference electrode installed in an undivided cell. Different potentials were investigated to establish the optimal reaction conditions. **1a** (0.2 mmol), *n*Bu_4_NOH (0.2 mmol), TBHP (1.0 mmol), and internal standard substance (*n*-nonane) were added into MeCN (10.0 ml) and stirred for 20 min, and the electrocatalytic depolymerization was carried out for 5 h under air. The mixture solution after electrocatalytic depolymerization was removed through a syringe with a 0.22-μm organic filter, followed by qualitative and quantitative analysis using GC-MS and GC.

### Characterizations

The morphologies of samples were characterized by SEM (JEOL, JSM-7500F, Japan). HR-TEM images, SAED, and EDS maps were taken by a JEOL TEM, JEM-F200 (Japan) operating at 200 kV. Powder x-ray diffraction was conducted in the 2*θ* range of 10° to 90° with Cu-Kα radiation (Rigaku SmartLab SE, Japan). XPS was carried out on a Thermo Fisher Scientific K-Alpha instrument (USA) with Al-Kα x-rays (1,489.6 eV, 150 W, 50.0-eV pass energy) using the C 1s peak at 284.8 eV as the internal standard. The chemical composition was detected by inductively coupled plasma-optical emission spectrometry (Thermo Fisher Scientific iCAP PRO, USA). Raman spectra were measured on a confocal microscope (HORIBA Scientific LabRAM HR Evolution, Japan) equipped with a semiconductor laser (*λ* = 532 nm). The synchrotron-based hard XAFS measurements were performed with Si(111) crystal monochromators at the BL14W1 beamlines at Shanghai Synchrotron Radiation Facility in China.

The product yields of lignin model cleavage and lignin depolymerization were analyzed by GC-MS (Agilent 7890A-5975C, HP-5MS column, USA), and quantitative analysis were performed using GC (Agilent 8860, HP-5 column, USA). The organosolv, Kraft lignin, and depolymerization product fractions were characterized by ^1^H–^13^C, HSQC NMR (Bruker Advance III HD 500 MHz, Switzerland). GPC of lignin and depolymerization product fractions was conducted using tetrahydrofuran as the mobile phase on a Waters 1525 & Agilent PL-GPC220 (USA).

## Data Availability

The data that support the findings of this study are available from the corresponding author upon reasonable request.

## Supplementary Materials

Supplementary 1 Supplementary References Experimental Section Figs. S1 to S32 Tables S1 to S3
