# Supplementary material for: Electrocatalytic Lignin Valorization into Aromatic Products via Oxidative Cleavage of Cα−Cβ Bonds
Source: Research (Wash D C). 2023 Dec 15;6:0288. doi: 10.34133/research.0288 (PMC10726294; doi:10.34133/research.0288)
Supplement: Supplementary 1 — Supplementary References Experimental Section Figs. S1 to S32 Tables S1 to S3 [file research.0288.f1.docx]

**Supplementary Information**

**Electrocatalytic Lignin Valorization into Aromatic Products *via* Oxidative Cleavage of C_α_−C_β_ Bonds**

Jianing Xu^1^, Juan Meng^3^, Yi Hu^1^, Yongzhuang Liu^1^, Yuhan Lou^1^, Wenjing Bai^1^, Shuo Dou^1*^, Haipeng Yu^1*^, and Shuangyin Wang^2*^

1. Key Laboratory of Bio-based Material Science and Technology of Ministry of Education, Northeast Forestry University, Harbin, 150040, China.
2. State Key Laboratory of Chem/Bio-Sensing and Chemometrics, College of Chemistry and Chemical Engineering, Hunan University, Changsha, 410082, China.
3. School of Resources and Environmental Engineering, Jiangsu University of Technology, Changzhou, 213001, China.

^*^Address correspondence to: : [yuhaipeng20000@nefu.edu.cn](mailto:yuhaipeng20000@nefu.edu.cn) (H.Y.); [doushuo@nefu.edu.cn](mailto:doushuo@nefu.edu.cn) (S.D); and [shuangyinwang@hnu.edu.cn](mailto:shuangyinwang@hnu.edu.cn) (S.W).

**Contents**1. Experimental section
2. Supplementary Figures

3. Supplementary Tables

4. Supplementary References

**Experimental section**

**Characterizations.**

The morphologies of samples were characterized by SEM (JEOL, JSM-7500F, Japan). HRTEM images, SAED, and EDS maps were taken by a JEOL TEM, JEM-F200 (Japan) operating at 200 kV. Powder XRD was conducted in the 2*θ* range of 10−90° with Cu-Kα radiation (Rigaku SmartLab SE, Japan). XPS was carried out on a Thermo Scientific K-Alpha instrument (USA) with Al Kα X-rays (1489.6 eV, 150 W, 50.0 eV pass energy) using the C 1s peak at 284.8 eV as the internal standard. The chemical composition was detected by ICP-OES (Thermo Fisher ICAP PRO, USA). Raman spectra were measured on a confocal microscope (HORIBA Scientific LabRAM HR Evolution, Japan) equipped with a semiconductor laser (λ = 532 nm). The synchrotron-based hard X-ray absorption fine spectroscopy (XAFS) measurements were performed with Si(111) crystal monochromators at the BL14W1 beamlines at Shanghai Synchrotron Radiation Facility in China.

The product yields of lignin model cleavage and lignin depolymerization were analyzed by gas chromatography-mass spectrometry (GC-MS; Agilent 7890A-5975C, HP-5MS column, USA), and quantitative analysis were performed using gas chromatography (GC; Agilent 8860, HP-5 column, USA). The organosolv, Kraft lignin, and depolymerization product fractions were characterized by ^1^H-^13^C, HSQC NMR (Bruker Advance III HD 500 MHz, Switzerland). Gel permeation chromatography (GPC) of lignin and depolymerization product fractions was conducted using THF as the mobile phase on a Waters 1525 & Agilent PL-GPC220 (USA).

**Synthesis of** **1T-MoS_2_ template.**

The synthesis of 1T-MoS_2_ nanosheets was carried out through a hydrothermal method. Ammonium molybdate tetrahydrate [(NH_4_)_6_Mo_7_O_24_·4H_2_O] and thiourea (CH_4_N_2_S) were added into deionized water and stirred 1 h to dissolve thoroughly. Then, HCl was added to adjust the pH to 3 and stirred for 30 minutes. The solution was transferred into a Teflon-line stainless-steel autoclave, and a piece of carbon paper was immersed in the solution as substrate. The autoclave was heated to 180 ºC for 24 h and cooled to room temperature naturally. The obtained samples were washed with ultrapure water and ethanol to remove excess reactant and dried at 60 ºC in a vacuum^1, 2^.

**Synthesis of** **Mo@Ni_9_Co_1_OOH (MoNiCo (oxy)hydroxides).**

The MoS_2_/NiCo LDH pre-catalysts were constructed by a chemical bath. MoS_2_ nanosheets were activated at -1.5 V (vs. Ag/AgCl) in 1M KOH to improve the hydrophilia and then washed with deionized water thoroughly. The resulting samples were further immersed in a mixed solution of NiCl_2_·6H_2_O and Co(NO_3_)_2_·6H_2_O (30 mM) for 20 min for physically adsorbing Ni and Co ions on the surface to construct the pre-catalysts. The doping ratio of two metals can be precisely regulated in this process. Then, the pre-catalysts were washed with deionized water thoroughly to remove excess adsorbate and dried at ambient conditions. After that, the MoS_2_/NiCo LDH pre-catalysts were subjected to 5 cycles of CV activation (the potential range from 0 V to 0.8 V (vs. Ag/AgCl) with a scan rate of 5 mV/s) in 1 M KOH solution to obtain self-reconstruction Mo doping MoNiCo (oxy)hydroxide through Mo leaching^2^.

**Synthesis of Mo@NiOOH (MoNi (oxy)hydroxides).**

MoNi (oxy)hydroxide and were synthesized in the same method except for without using Co(NO_3_)_2_·6H_2_O.

**Synthesis of pure Ni_9_Co_1_OOH.**

Typically, 2.7 mmol NiCl_2_·6H_2_O and 0.3 mmol Co(NO_3_)_2_·6H_2_O with 9 mmol urea were dissolved in 60 mL of distilled water and stirred for 20 minutes to dissolve thoroughly. The solution was subsequently transferred into a 100 mL Teflon-line stainless-steel autoclave, and a piece of carbon paper was immersed in the solution as substrate. The autoclave was heated to 120 ºC for 24 h and cooled to room temperature naturally. The obtained samples were washed with ultrapure water and ethanol to remove excess reactant and then dried at 60 ºC in a vacuum^3^.

**Electrocatalytic depolymerization of lignin model compound.**

Electrochemical measurements were performed using a three-electrode system in a CHI-660E electrochemical station. MeCN was used as the solvent with the addition of *n*Bu_4_NOH (1.0 M MeOH solution) to constitute the organic electrolyte. Mo@Ni_9_Co_1_OOH was used as the working electrode with a Pt counter electrode and Ag/AgCl reference electrode installed in an undivided cell. Different potentials were investigated to establish the optimal reaction conditions. **1a** (0.2 mmol), *n*Bu_4_NOH (0.2 mmol), TBHP (1.0 mmol), and internal standard substance (*n*-nonane) were added into MeCN (10.0 mL) and stirred for 20 minutes, and the electrocatalytic depolymerization was carried out for 5h under air. The mixture solution after electrocatalytic depolymerization was removed through a syringe with a 0.22 μm organic filter followed by qualitative and quantitative analysis using GC-MS and GC.

**Extraction of** **organosolv lignin and depolymerization measurements.**

Organosolv lignin from poplar was isolated by 1,4-dioxane in a conventional Soxhlet apparatus. 10 g of powdered biomass was placed in filter paper and inserted into a thimble holder within the Soxhlet apparatus. A 200 mL solution containing 1,4-dioxane, 0.4% v/v of concentrated HCl, and 3% v/v distilled water was used as the solvent mixture, which was added from the top and heated gradually in the distillation flask until the extraction chamber showed no further release of color. The solution was then concentrated using a rotary evaporator, and lignin was precipitated from the concentrate by adding lukewarm water and a small amount of ammonium chloride. Finally, the precipitate was filtered through a vacuum setup and dried in an oven to obtain the organosolv lignin.

The electrocatalytic depolymerization of lignin was similar to that of the lignin model compound. After the reaction, the solvent mixture was concentrated by a rotary evaporator at 35 ^o^C to remove the solvent, and the residue was extracted with ethyl acetate for further GC, GC-MS, GPC, and 2D HSQC NMR analysis.

**Calculation of products from lignin model and lignin material cleavage.**

**Lignin model : ^4, 5^**

Yield of product monomer **=** $\frac{\text{mole of monomer}}{\text{mole of lignin model}}$×100%

C_α_-C_β_ bond cleavage yield **=** $\frac{\text{moles of }\text{2a}\text{+ }\text{3a}}{\text{mole of lignin model}}$×100%

Conversion of lignin model **=** $\text{(}\text{1}\text{-}\frac{\text{mole of residual }\text{1a}}{\text{mole of lignin model}}\text{ }\text{)}$×100%

C_α_-C_β_ bond cleavage selectivity **=** $\frac{\text{C}\text{α}\text{-C}\text{β}\text{ bond cleavage yield}}{\text{conversion of lignin model}}$×100%

The mole of every product was calculated by the internal standard method of GC.

**Organosolv lignin and Kraft lignin:**

Yield of product monomer (wt%) **=** $\frac{\text{mole of monomer × M}}{\text{mass of lignin }}$×100%

**M**: relative molecular mass;

The mole of every product was calculated by the external standard method of GC.

**Theoretical calculation.**

First-principle calculations were performed with the density functional theory (DFT) as implemented in Vienna ab-initio Simulation Package (VASP) package. The augmented wave (PAW) method was used for the interactions between ions and valence electrons with Perdew-Burke-Ernzerhof (PBE) functional. The plane wave energy cutoff was set to 400 eV. The atomic force and energy convergence criteria thresholds were set to 0.02 eV and 1×10^−5^ eV/Å, respectively. The *k*-point mesh of 2×2×1 was employed for the geometric optimization and density of states calculation. The van der Waals (vdW) interaction was taken into account with Grimme type at DFT-D3 level. The chemical reaction barrier along different pathways is calculated with climbing image nudged elastic band (dimer-method) method interfaced with VASP. The free energies of each structure were calculated at room temperature. The Ni-edge of NiOOH monolayers (105) were modeled as catalysis with Ni atoms linking on the MoS_2_ substrate.

**Supplementary Figures**


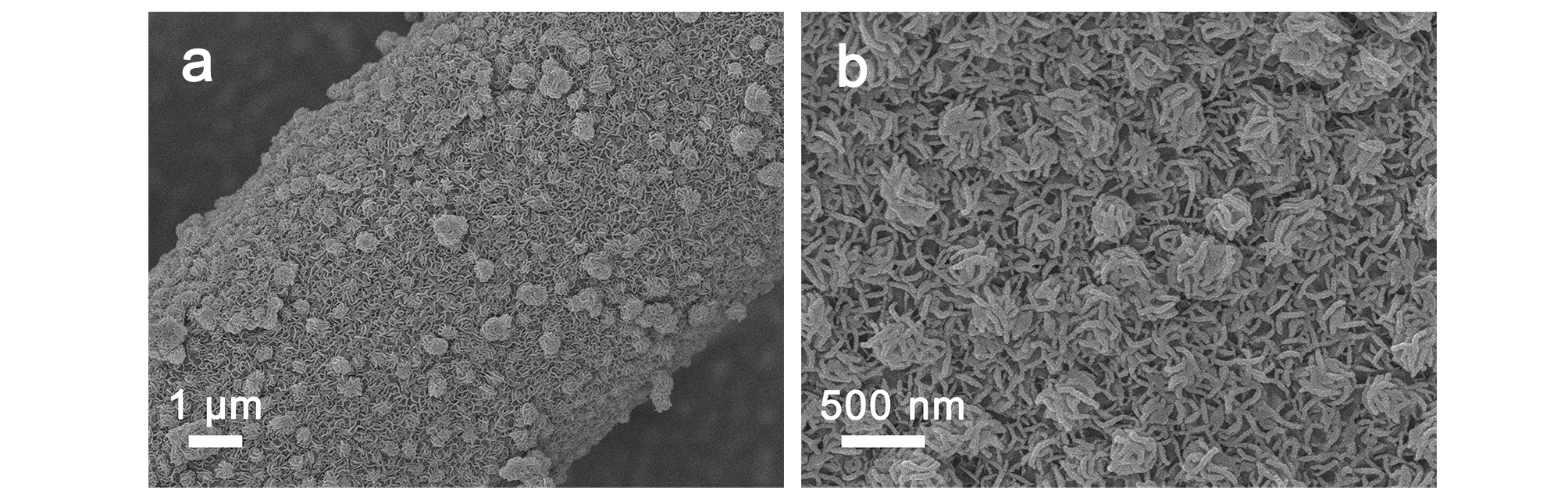


**Fig. S1.** Structure characterizations of the 1T-MoS_2_ nanosheets template. SEM images with (a) low magnification and (b) high magnification.


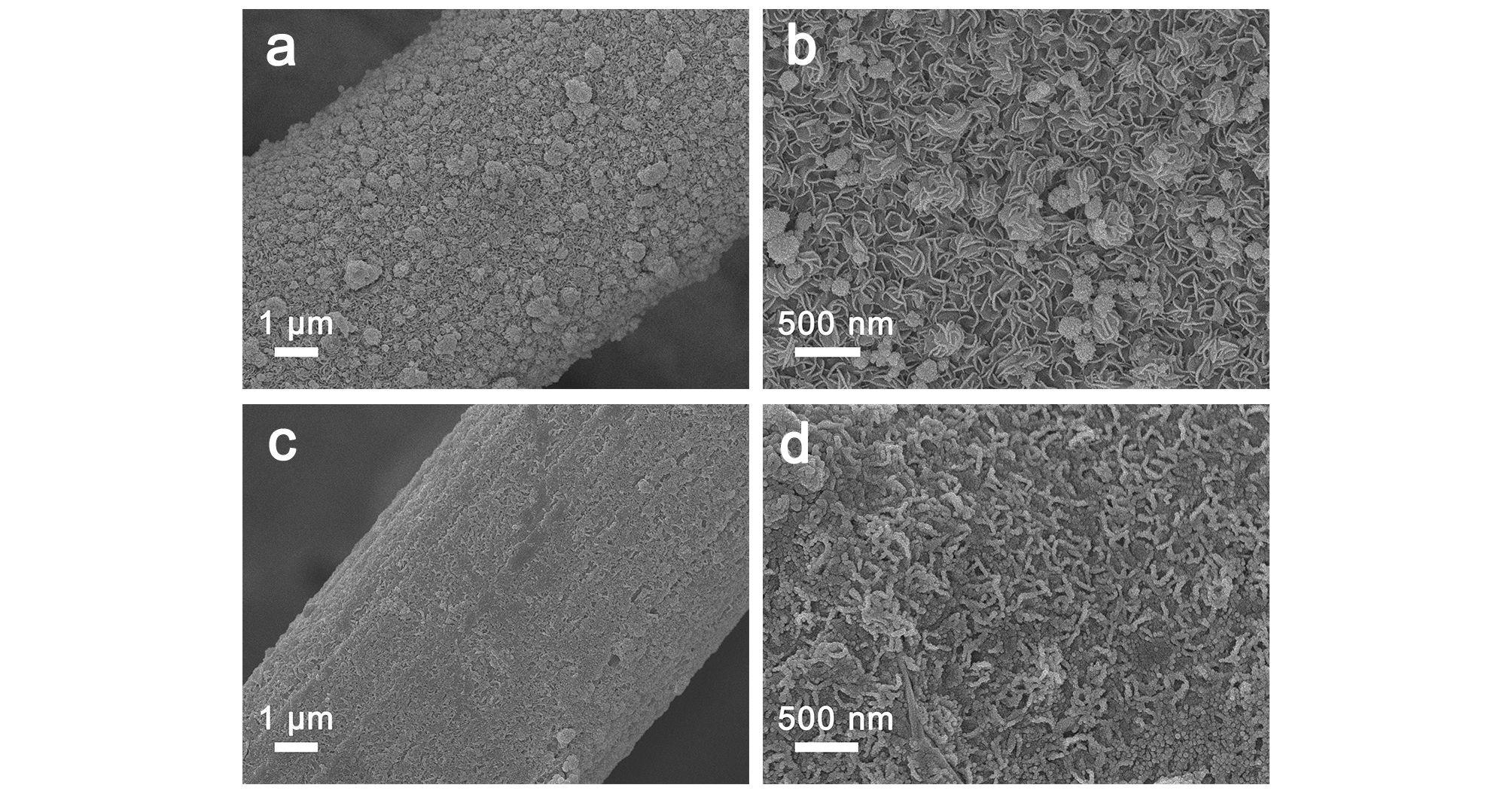


**Fig. S2.** Structure characterizations of the Mo@NiOOH. SEM images of Ni hydroxide-MoS_2_ (before activation) (a) low magnification and (b) high magnification. Mo@NiOOH (after activation) (c) low magnification and (d) high magnification.


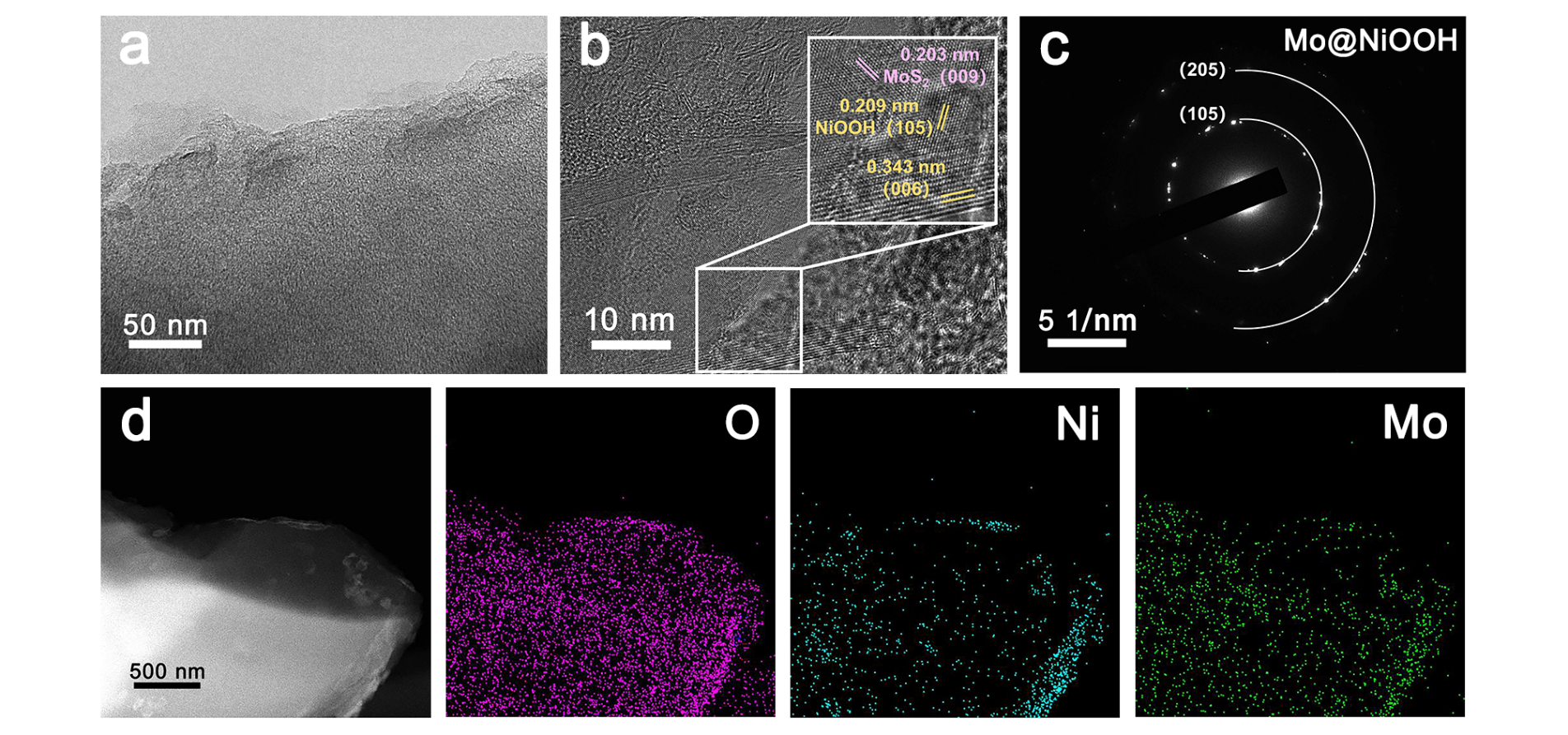


**Fig. S3.** TEM characterizations of the Mo@NiOOH. HRTEM images with (a) low magnification and (b) high magnification with lattice characteristics. (c) the SAED pattern. (d) the HRTEM image and EDS mapping.


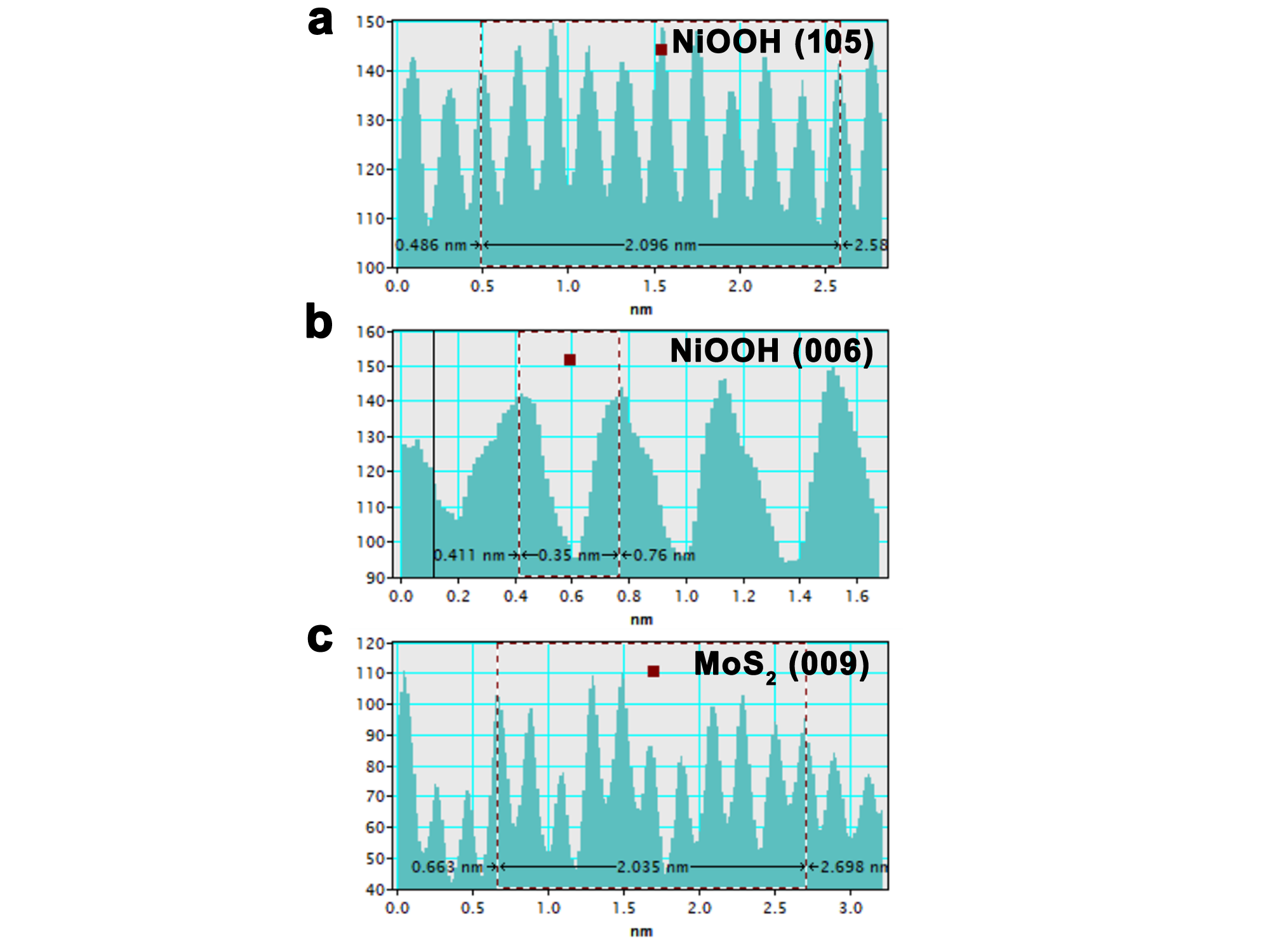


**Fig. S4.** The lattice spacing measured by Digital Micrograph fit in (a) NiOOH (105), (b) NiOOH (006) (c) MoS (009) for the magnifying area of Fig. S1 (f) and (g).


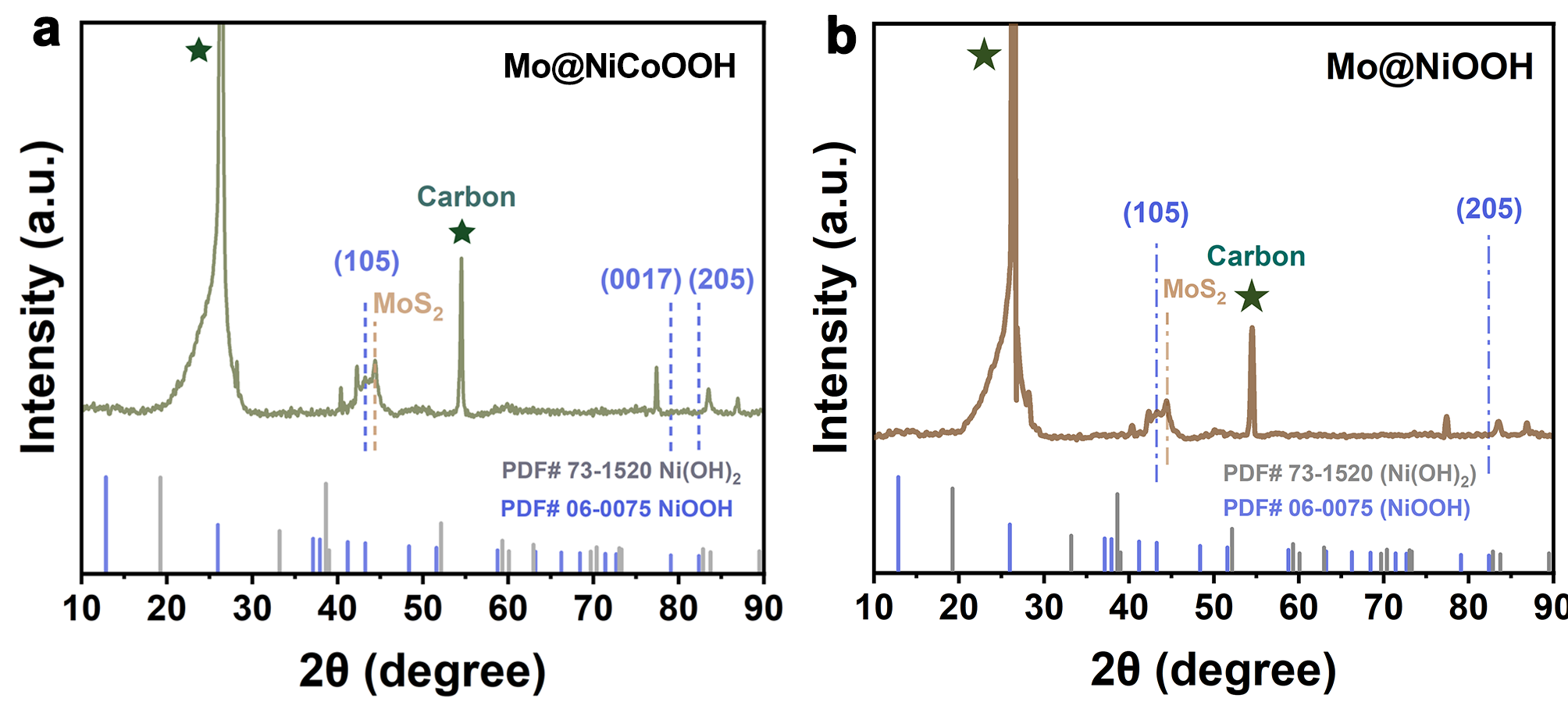


**Fig. S5.** Crystal structure characterizations. XRD patterns of (a) Mo@NiCoOOH and (b) Mo@NiOOH.


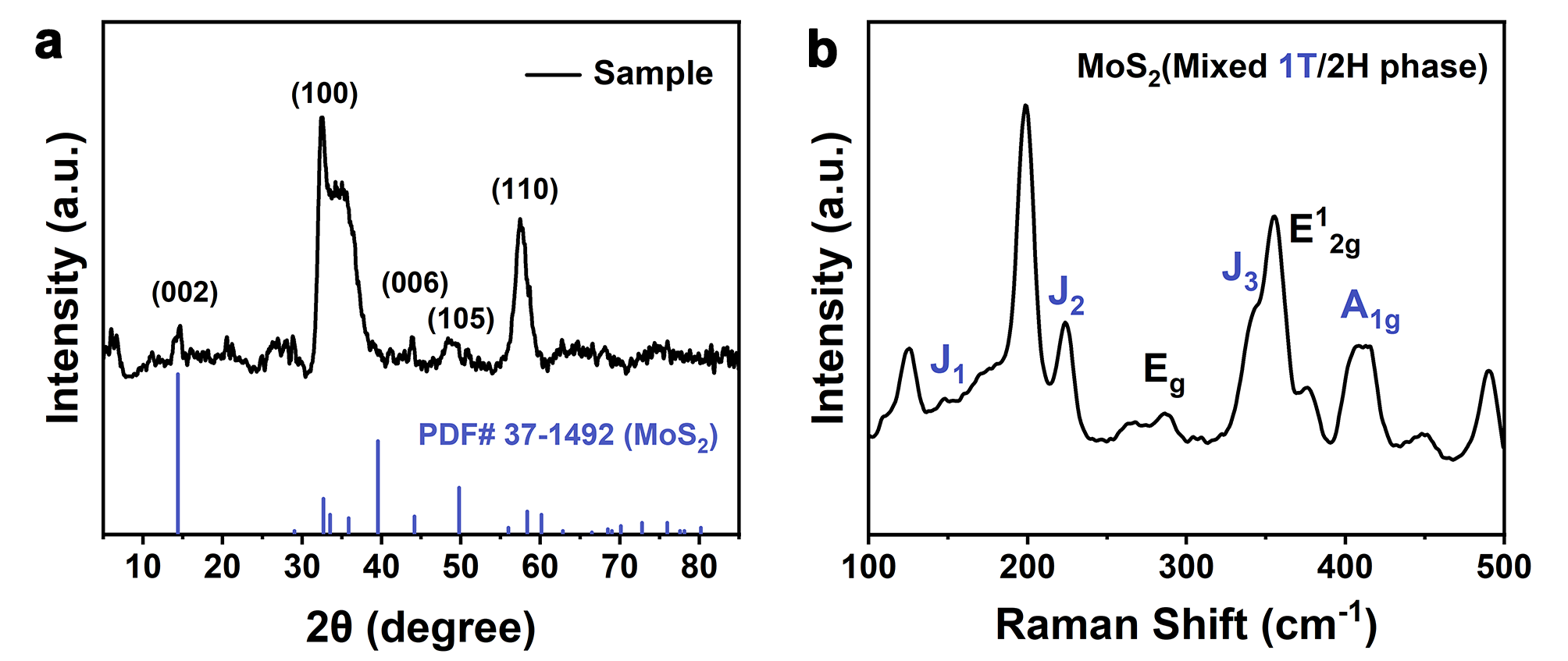


**Fig. S6.** Spectrochemical characterizations of MoS_2_. (a) The XRD pattern and (b) Raman spectrum of 1T-MoS_2_ template.


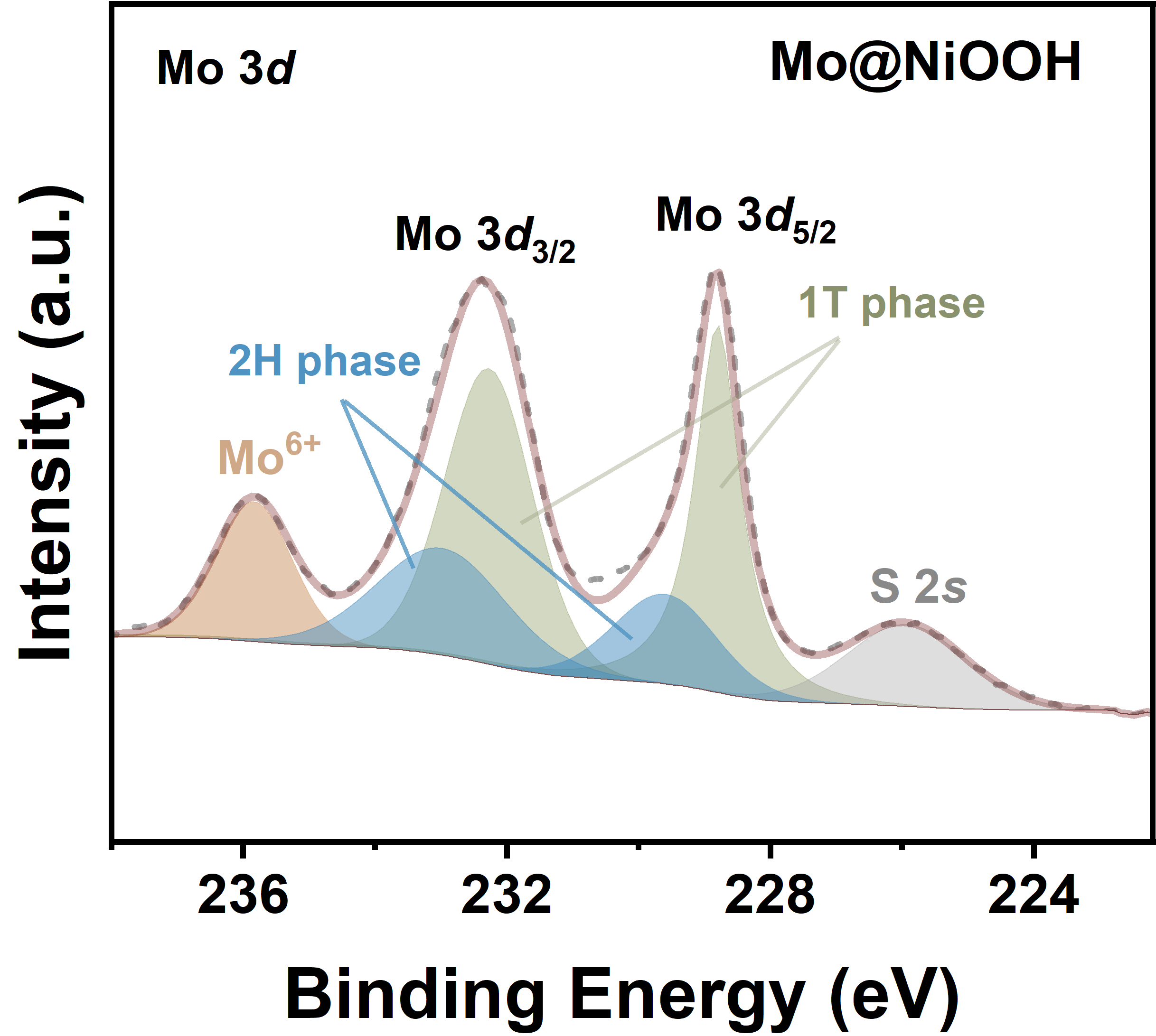


**Fig. S7.** XPS spectra in the Mo 3d of Mo@NiOOH.


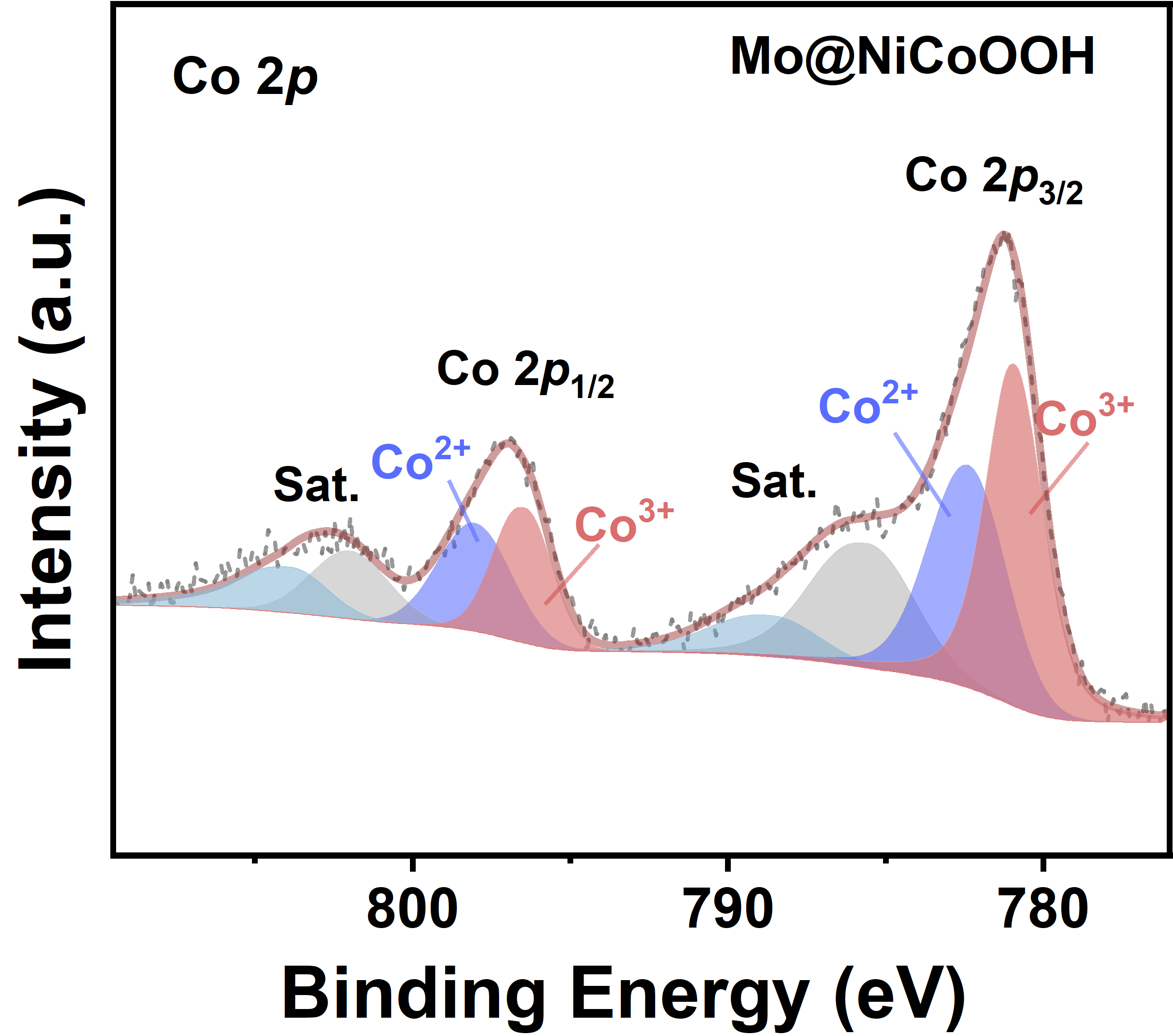


**Fig. S8.** XPS spectra in the Co 2p of MoNiCo (Oxy)hydroxide (Mo@Ni_9_Co_1_OOH).


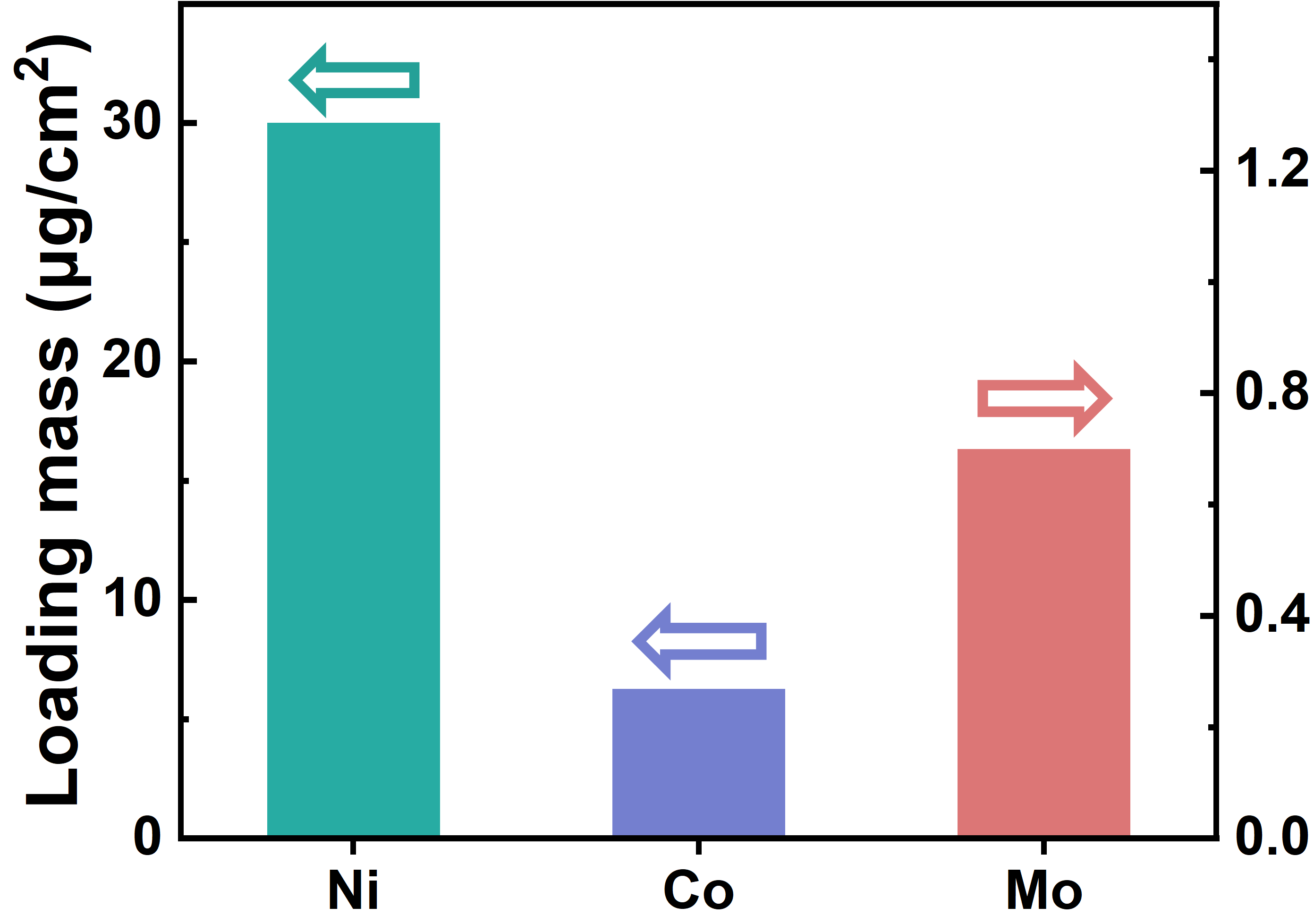


**Fig. S9.** Chemical composition characterizations for Mo@Ni_9_Co_1_OOH. The loading content of Ni, Co, Mo cation determined by ICP-OES measurement.


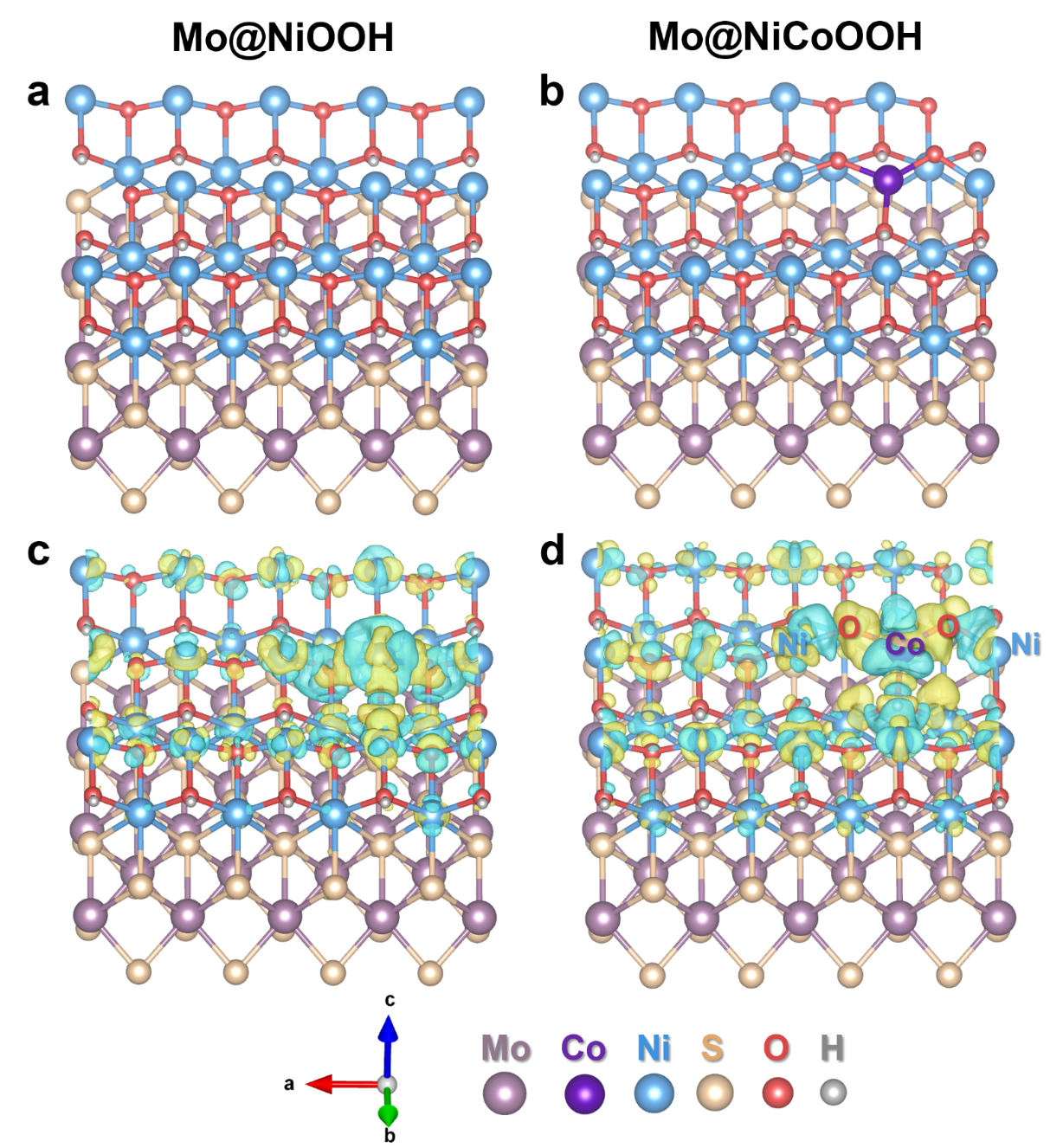


**Fig. S10.** 3D differential-charge density diagrams. Optimized structure models of (a) Mo@NiOOH and (b) Mo@NiCoOOH. For differential-charge densities of (c) Mo@NiOOH and (d) Mo@NiCoOOH, yellow and blue regions represent excess and depletion of charge density, respectively.


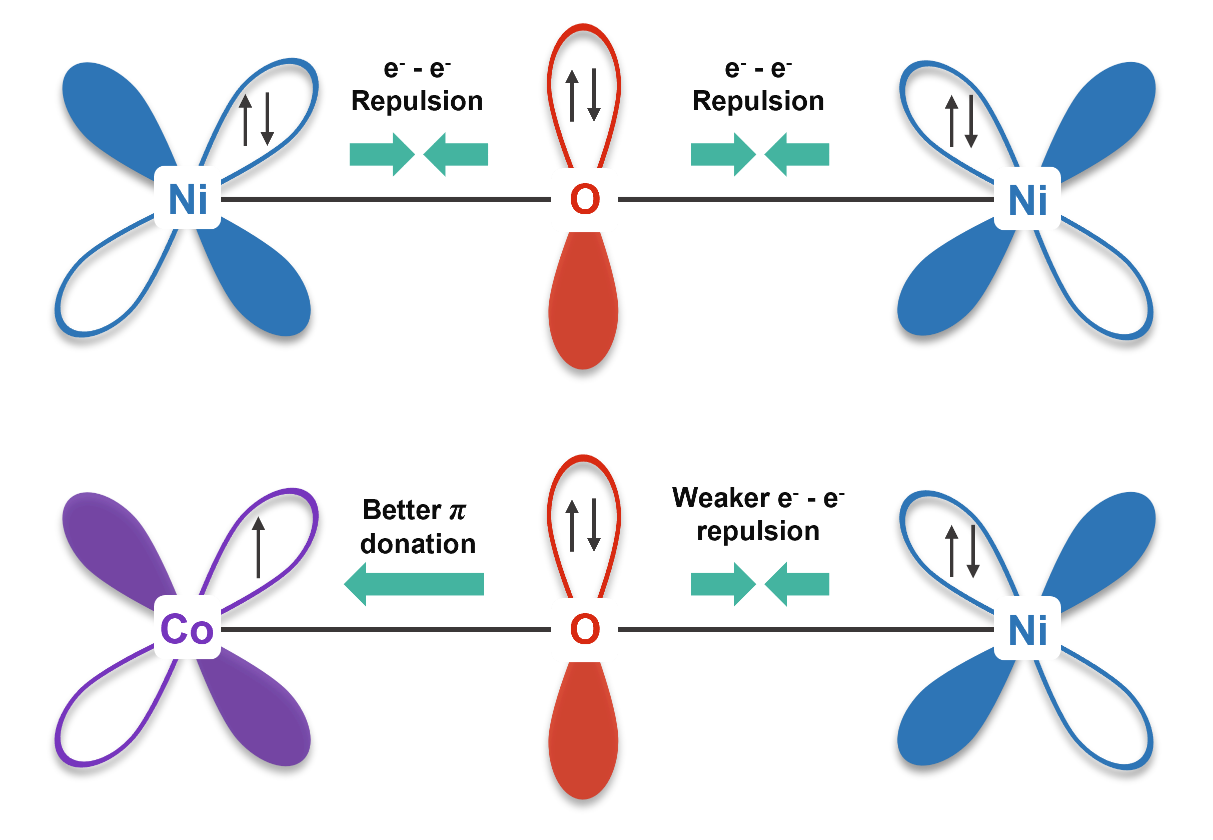


**Fig. S11.** Schematic representation of the electronic coupling between Ni and Co in Mo@NiOOH and Mo@NiCoOOH


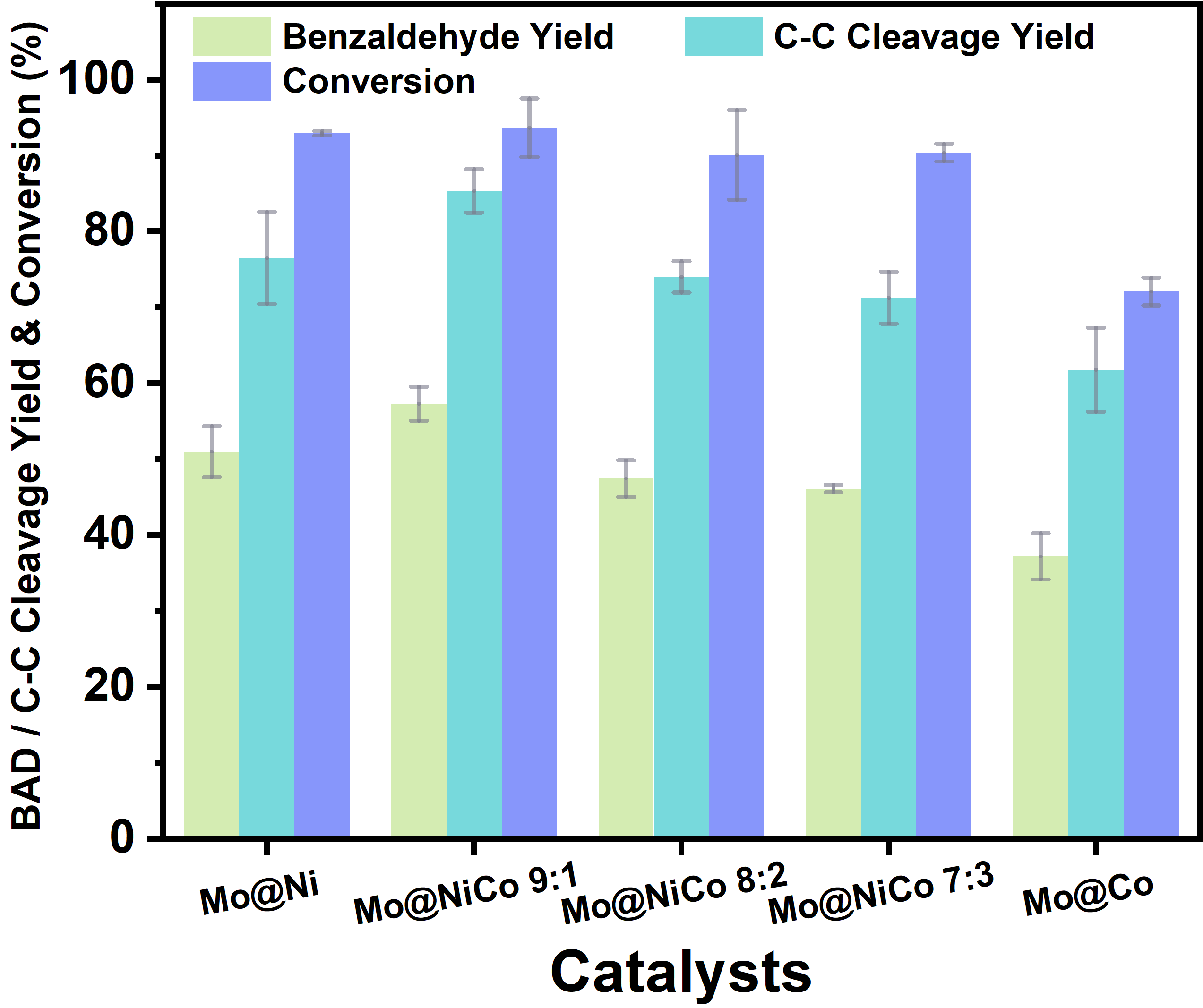


**Fig. S12.** The yield of mainly product **1b**, C_α_-C_β_ bonds cleavage and conversion of **1a** by different catalysts.


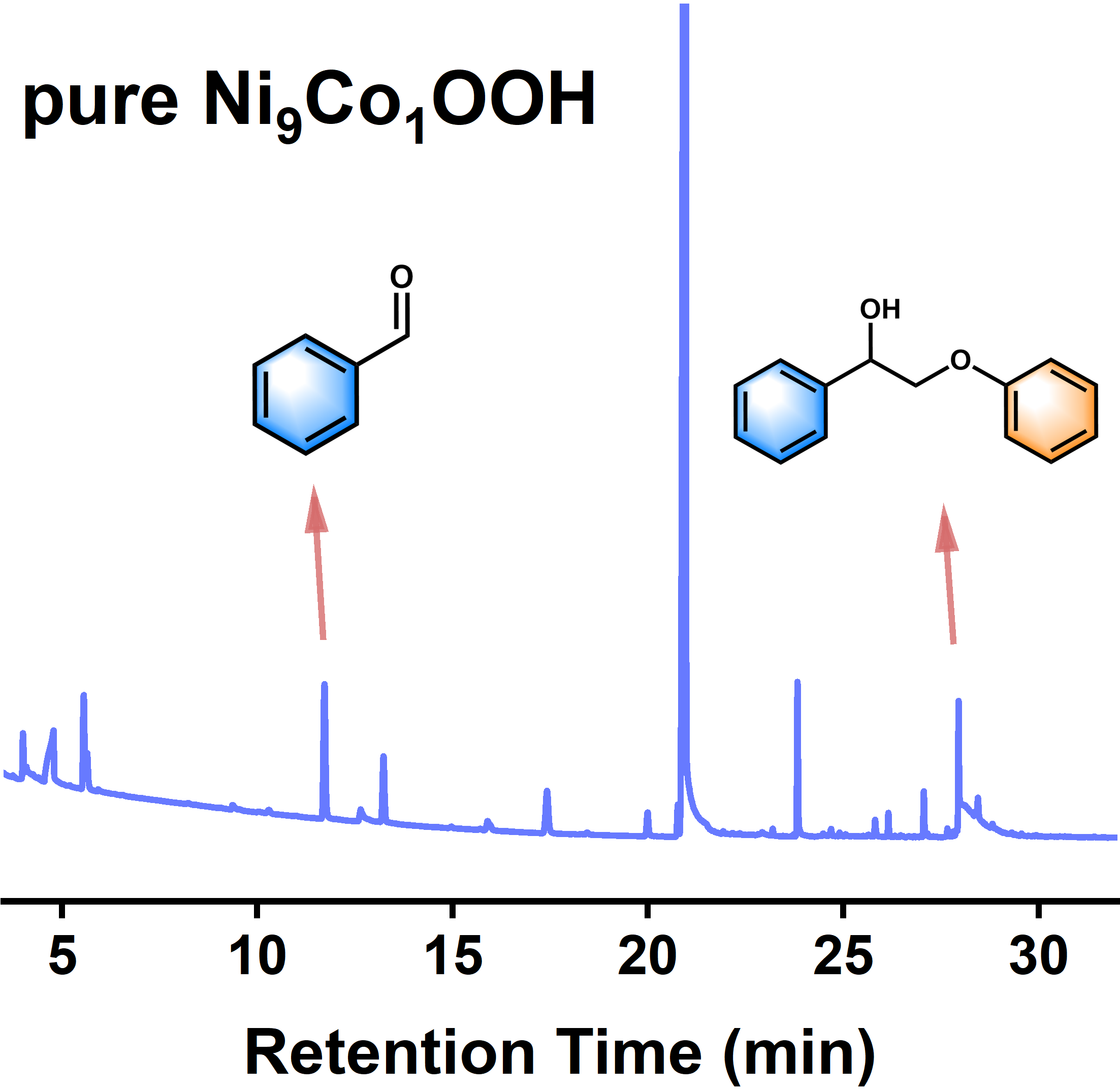


**Fig. S13.** GC chromatograms of the electrocatalytic oxidative reaction solution on Ni_9_Co_1_OOH catalyst under standard reaction conditions.


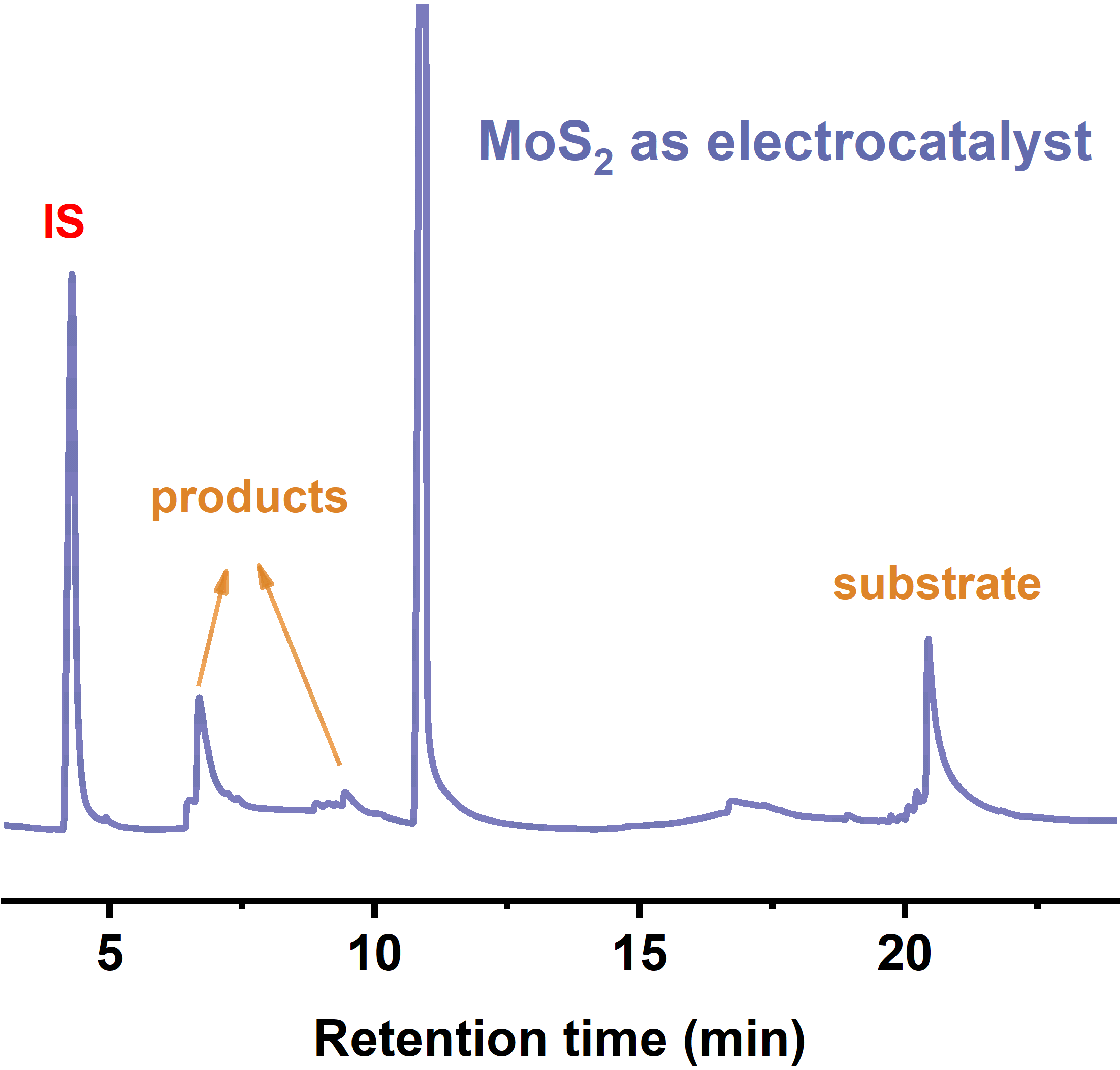


**Fig. S14.** GC chromatograms of the electrocatalytic oxidative reaction solution on pure MoS_2_ template under standard reaction conditions.


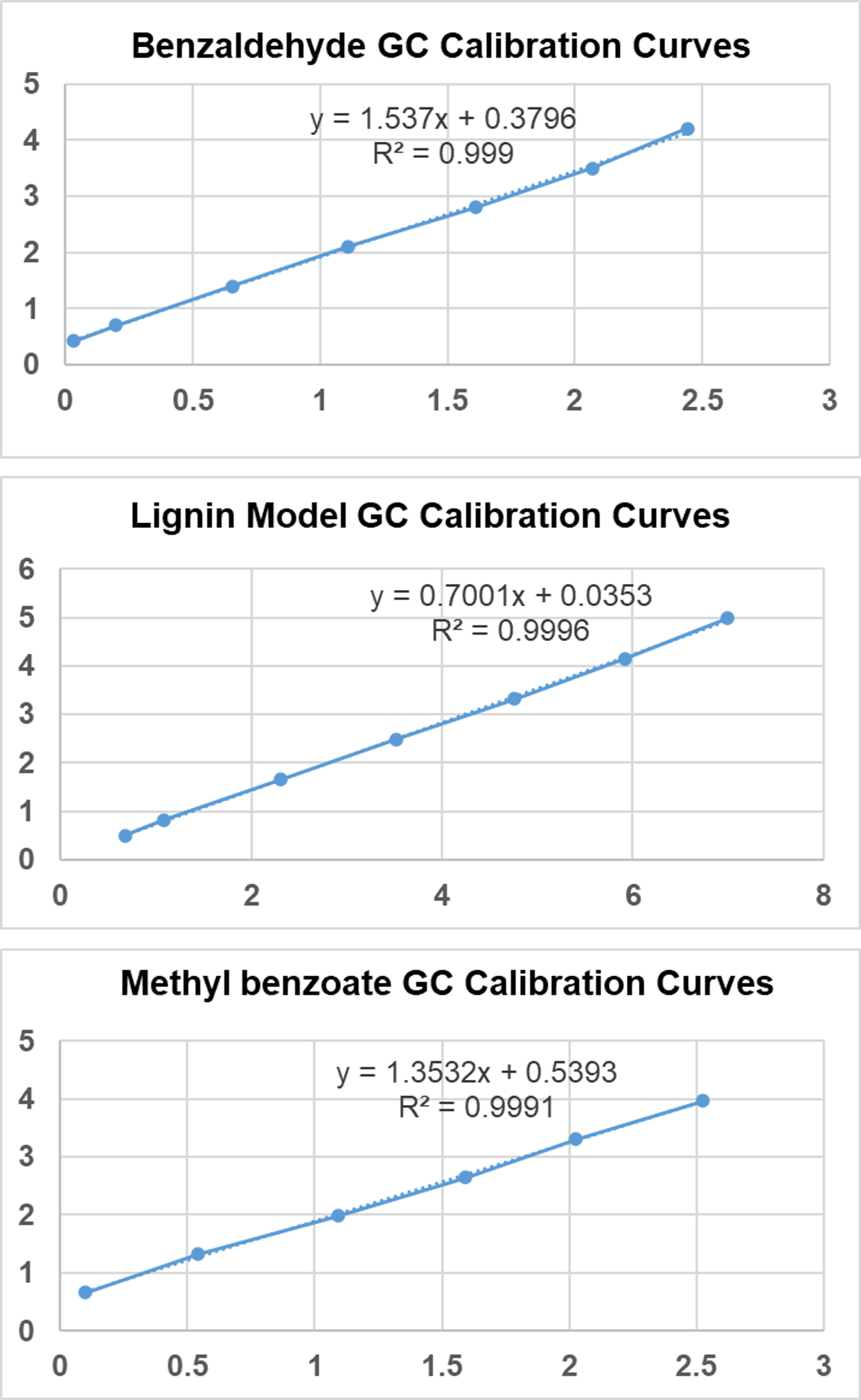


**Fig. S15.** Representative GC calibration curves for compounds identified from the depolymerization of lignin model. (Internal standard substance: n-Nonane)


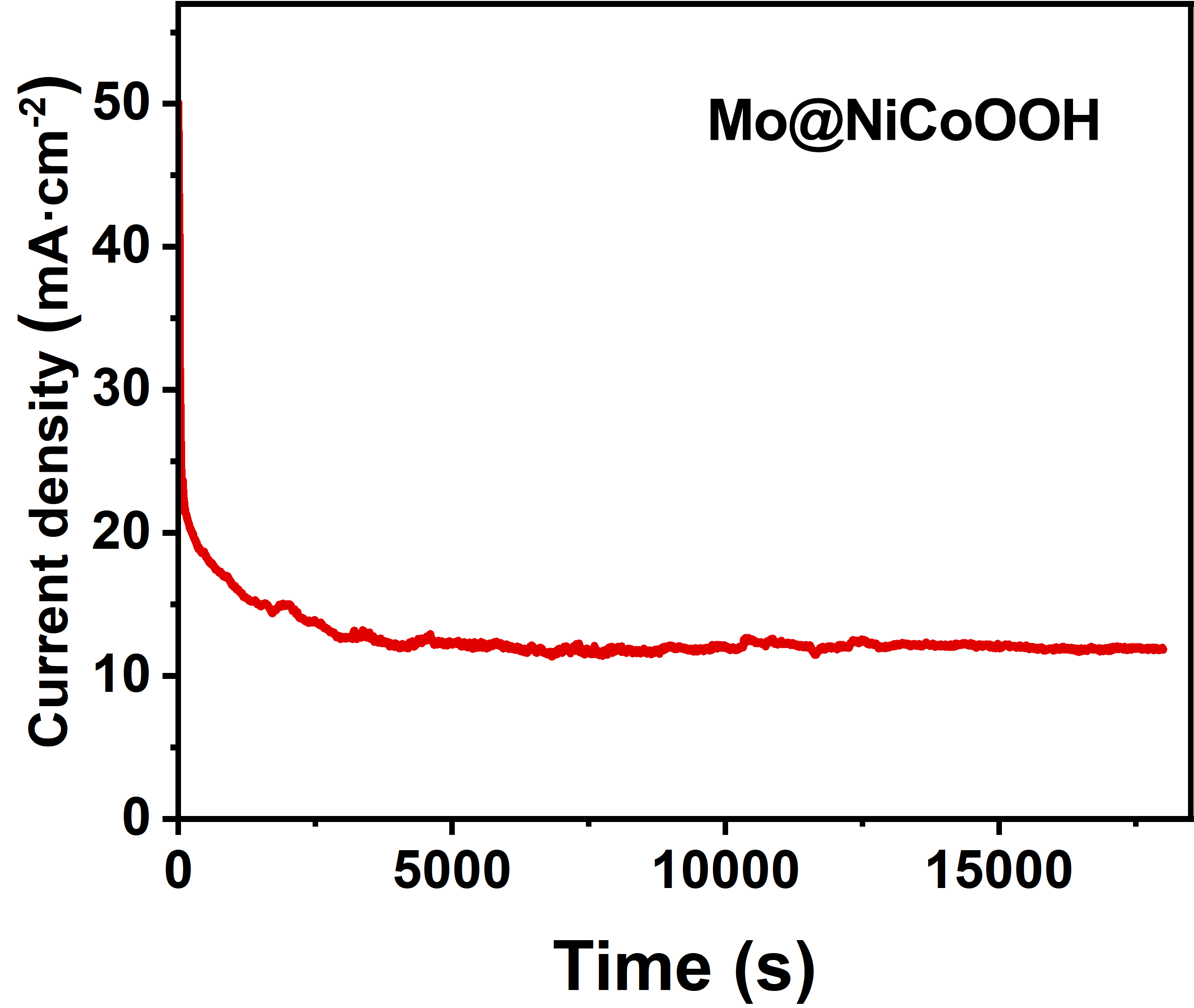


**Fig. S16.** The I-t curve of Mo@NiCoOOH for lignin model under standard reaction condition: **1a** (0.2 mmol), *n*Bu_4_NOH (0.2 mmol), TBHP (1.0 mmol), MeCN (10.0 mL), Internal standard (IS), RT, *E* = 3.5 V *vs*. Ag/AgCl, 5 h, under air.


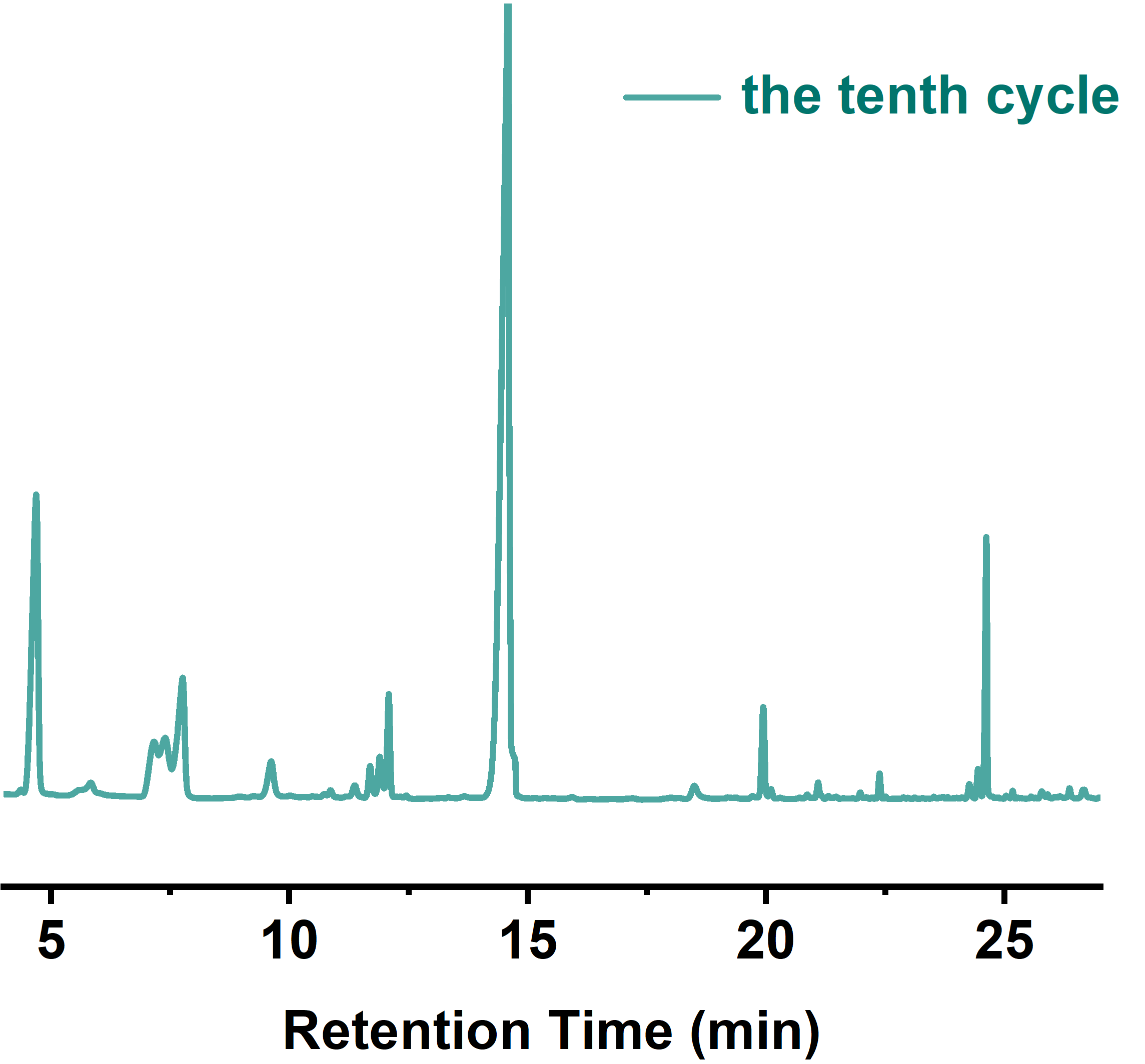


**Fig. S17.** GC chromatograms of the tenth electrocatalytic oxidative reaction cycle solution.


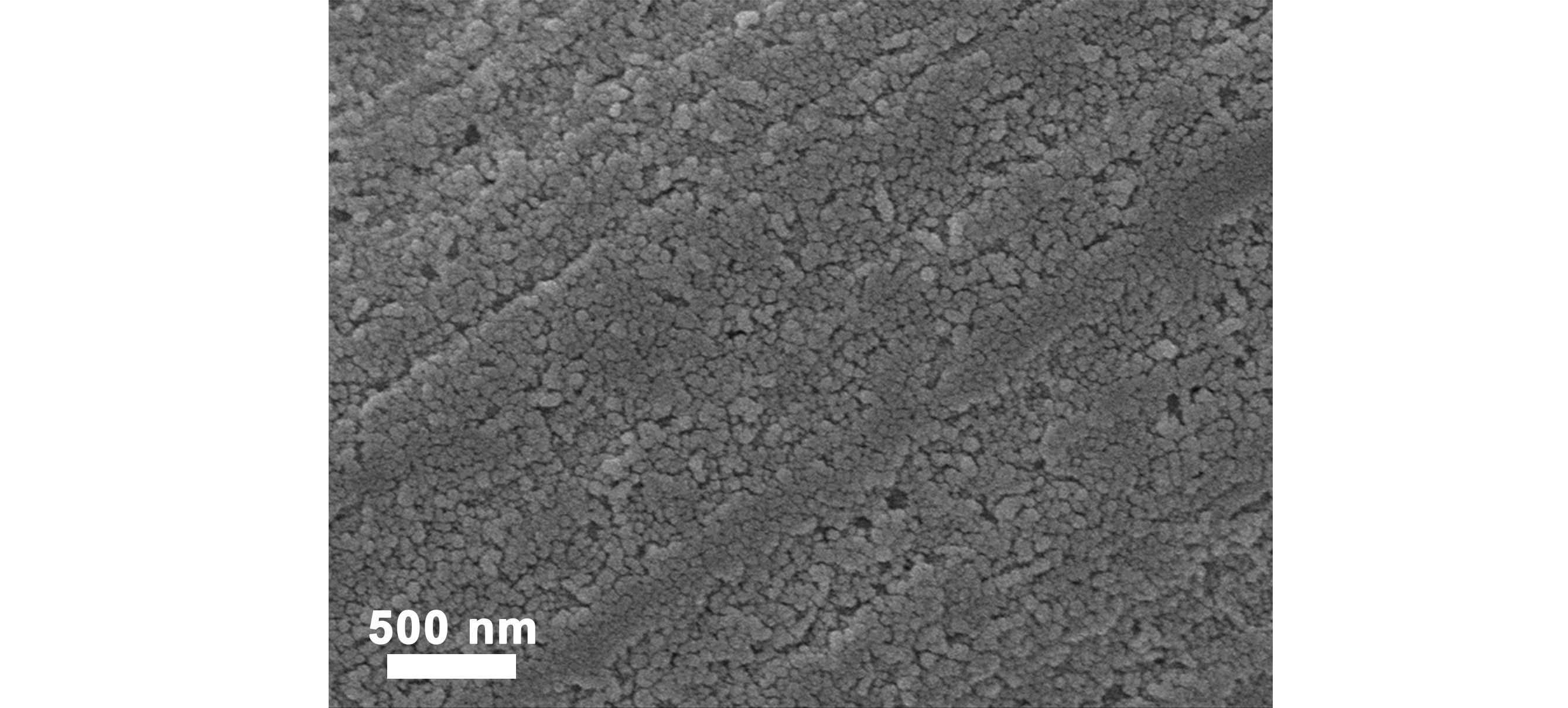


**Fig. S18.** SEM images of Mo@NiCoOOH heterojunction catalyst after ten cycles.


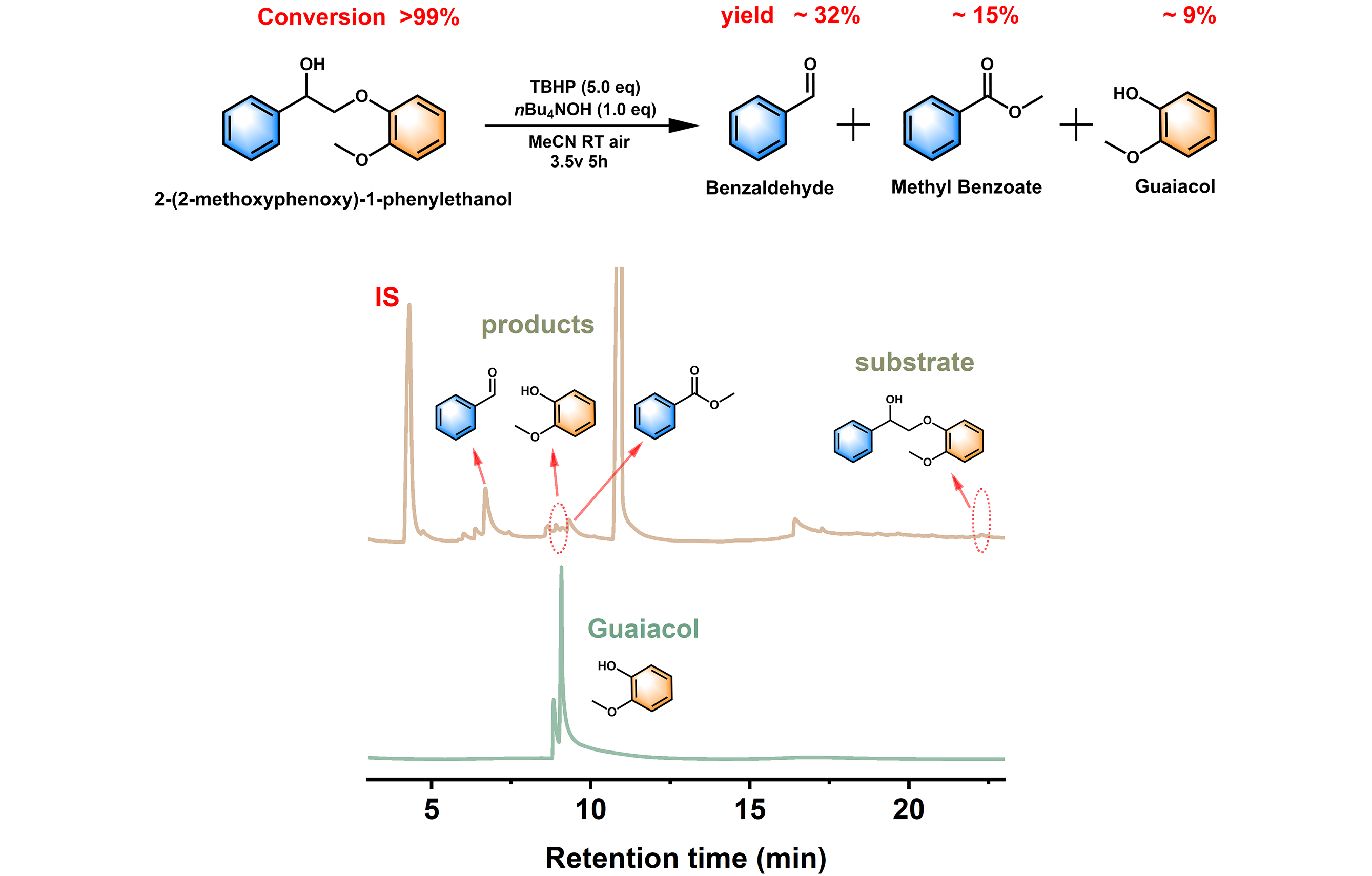


**Fig. S19.** Schematic and GC chromatograms of the oxidative cleavage reaction for other substrate (2-(2-Methoxyphenoxy)-1-phenylethanol) under standard reaction conditions.


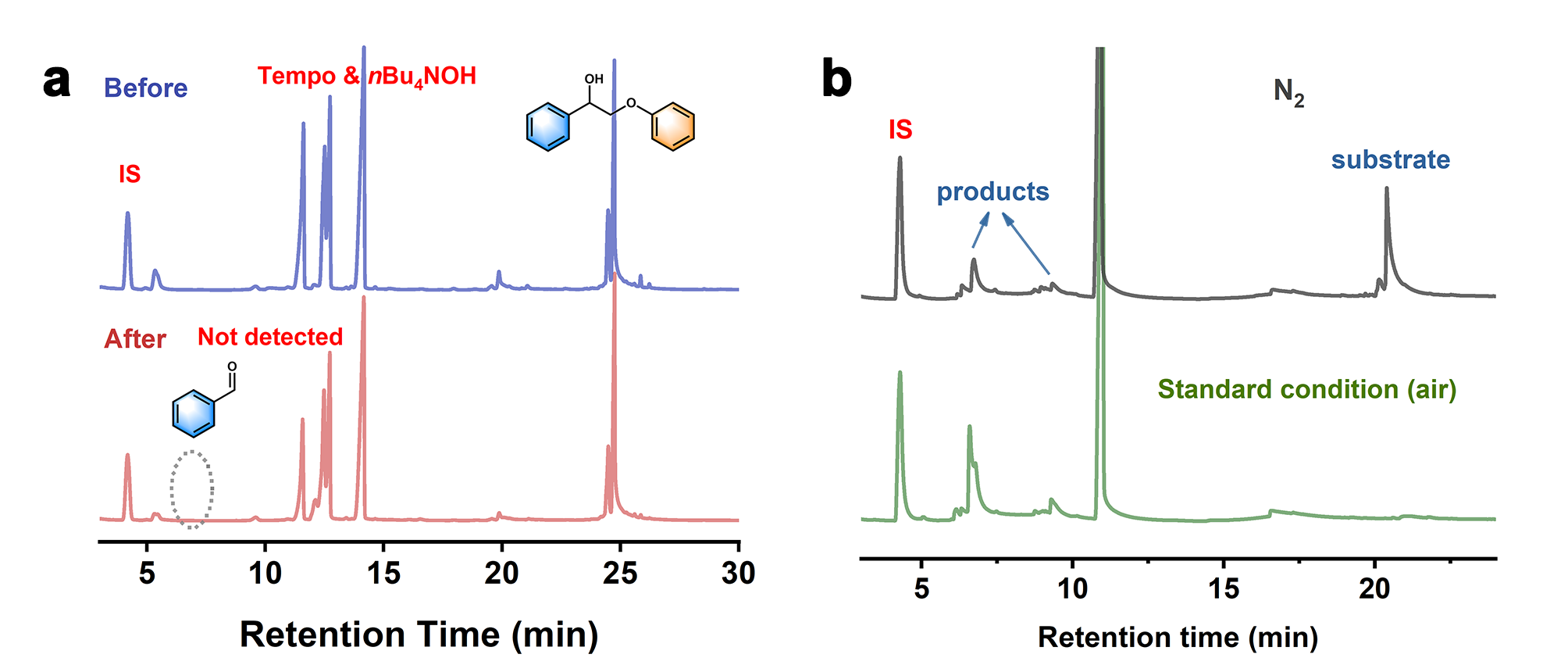


**Fig. S20.** Mechanism exploration. GC chromatograms comparison before and after electrocatalytic oxidative reaction with (**a**) radical scavenger (TEMPO) was added into the standard reaction mixture and (**b**) GC spectra comparison of N_2_ atmosphere and standard reaction conditions (ambient ).


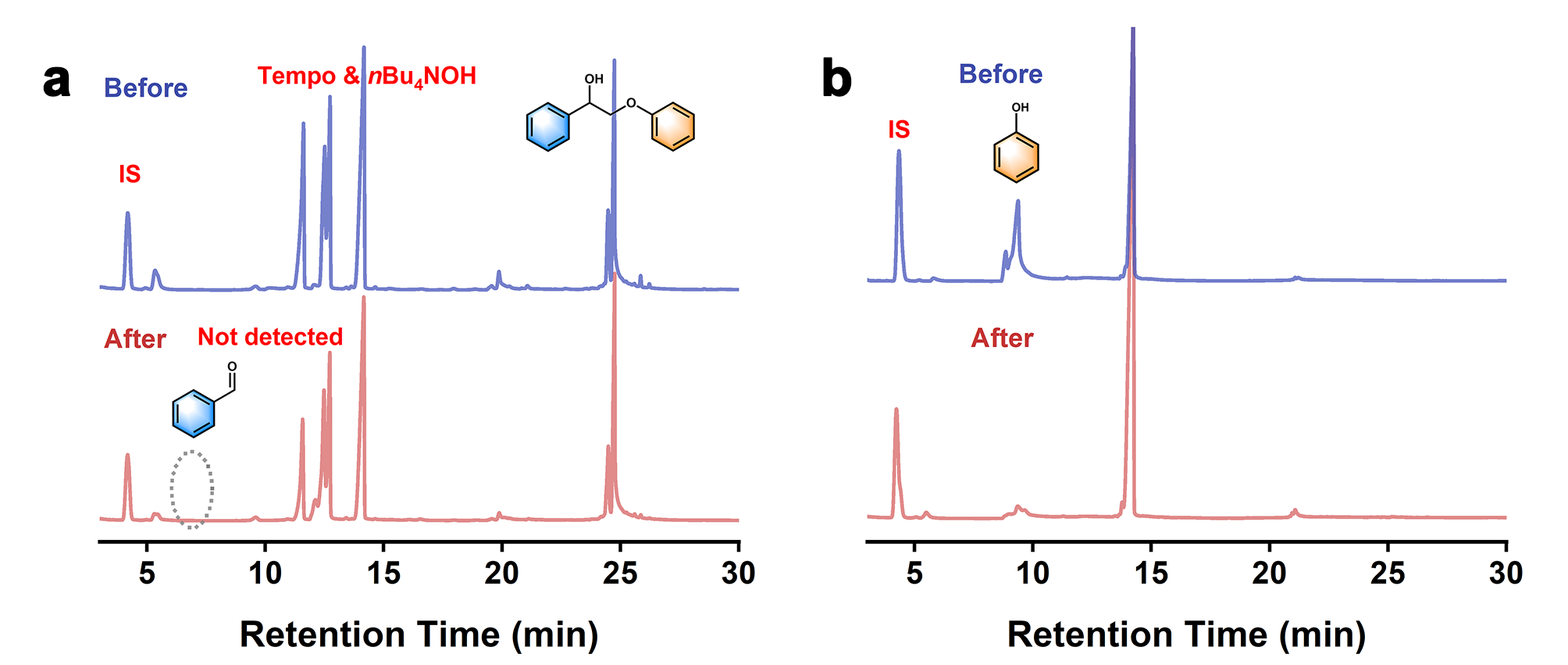


**Fig. S21.** Phenol as a substrate instead of **1a** under standard reaction condition.


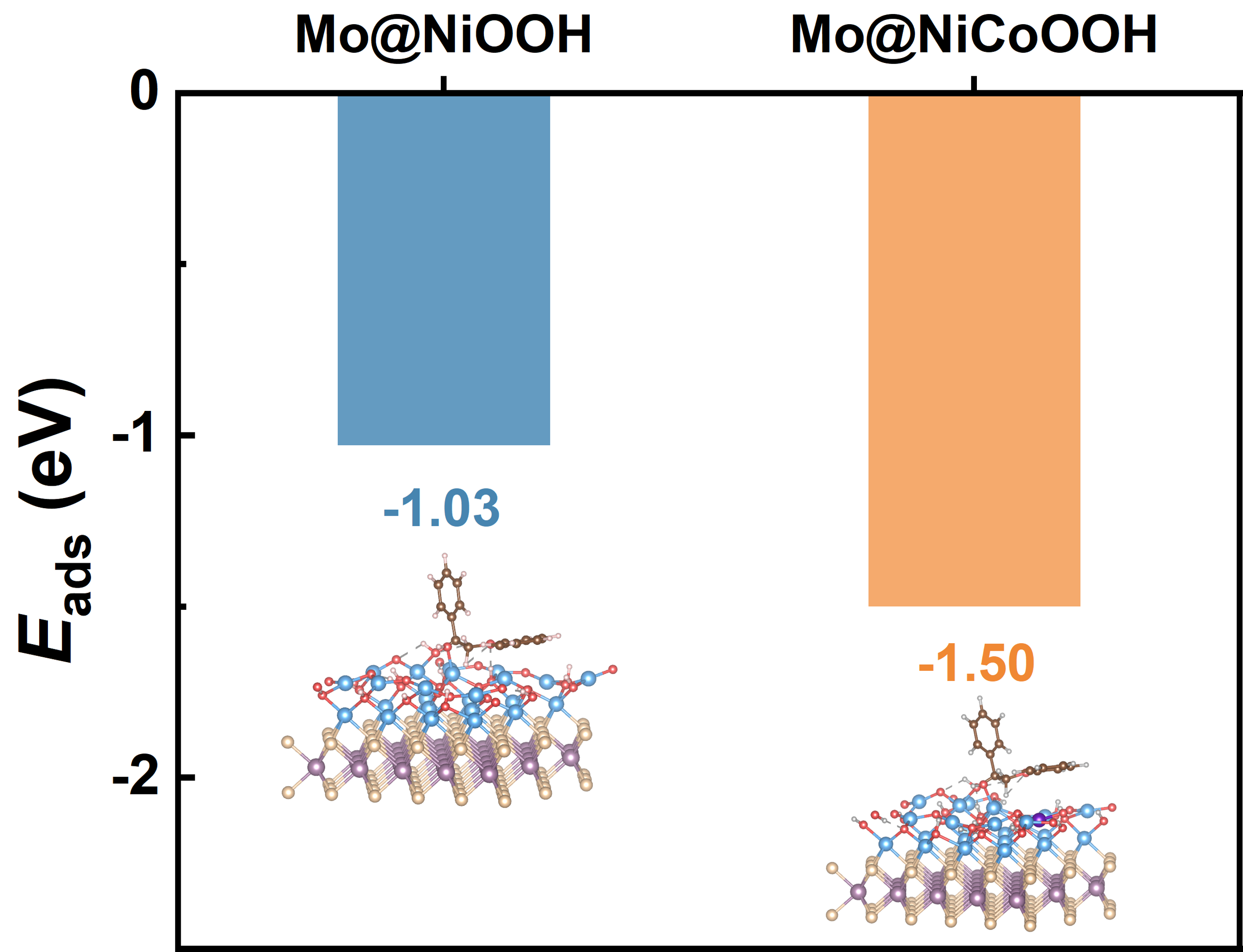


**Fig. S22.** Adsorption abilities. Optimized structures and adsorption energies of **1a** on Mo@NiOOH and Mo@NiCoOOH with OH^*^.


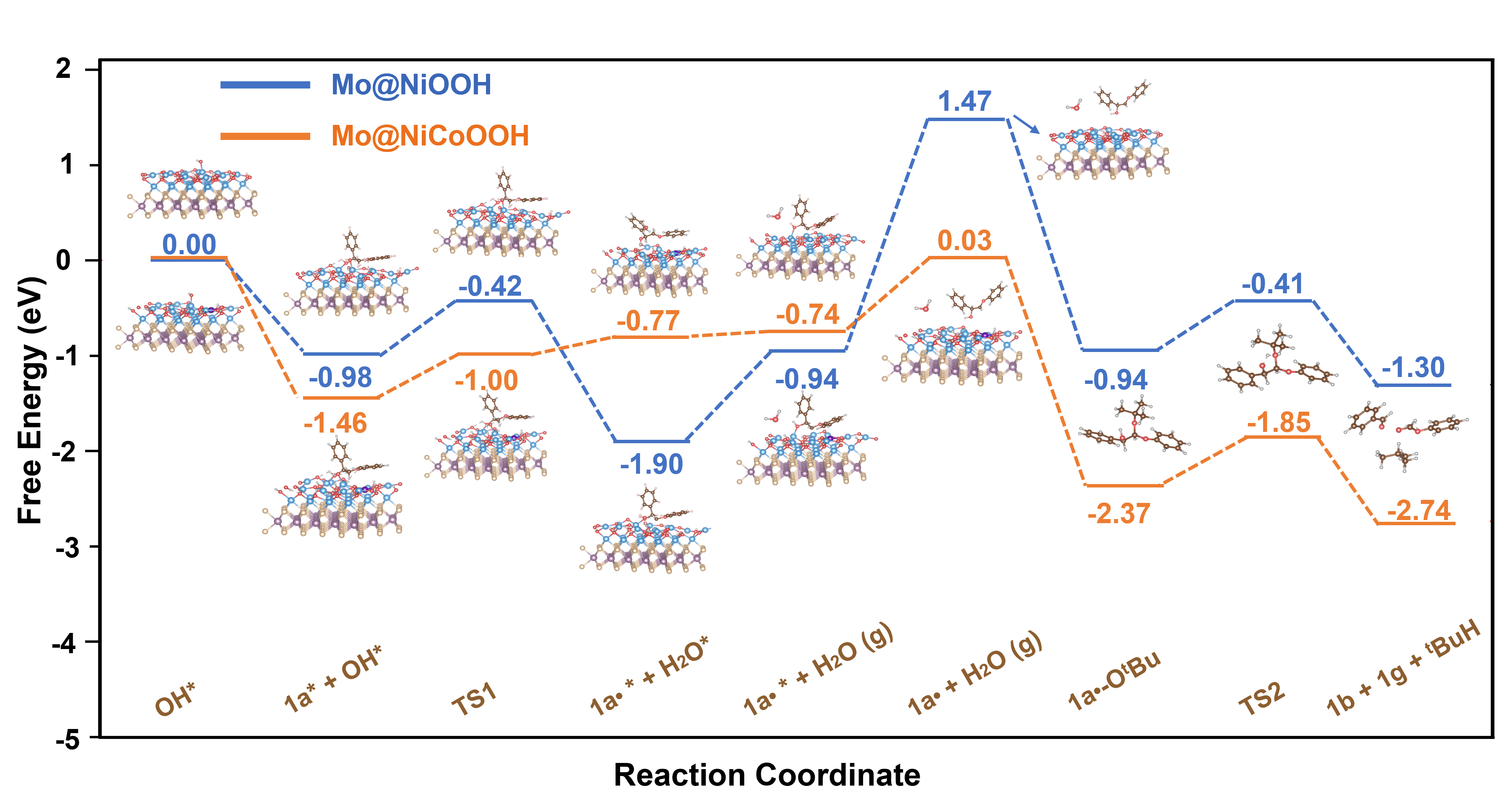


**Fig. S23**. The DFT-calculated Gibbs free energy diagram and optimized reaction intermediate structures for **1a** conversion on the Ni active sites of Mo@NiOOH and Mo@NiCoOOH in route 2. (In the case of Mo@NiCoOOH, the first step involves the adsorption of **1a** on Ni site with a Gibbs free-energy change (ΔG) of −1.46 eV. Then, the H atom from **1a** transferred to the OH^*^ on the surface to form H_2_O^*^ and intermediate **1a’**^*^, the reaction energy and energy barrier for this step were calculated to be 0.22 and 0.46 eV, respectively. Subsequently, the H_2_O^*^ was desorbed from the surface of catalyst (ΔG=0.50 eV), followed by the desorption of **1a’**^*^ as the rate-determining-step (RDS) with a ΔG of 0.77 eV. The generated **1a’** coupled with *^t^*BuO**·** to form **1a’-O*^t^*Bu** intermediate as an exothermic process of −2.40 eV. Ultimately, the compounds of **1b**, **1g,** and ***^t^*BuH** were produced *via* C_α_−C_β_ bond cleavage of **1a’-O*^t^*Bu**, with a reaction energy and energy barrier of −0.37 and 0.52 eV. Note that the pathway for Mo@NiOOH exhibited the same RDS for **1a’**^*^ desorption but had a higher ΔG of 2.41 eV, which indicated **1a’**^*^ had difficulty participating in the following reaction, thus hindering the overall efficiency of C_α_−C_β_ bond cleavage. Moreover, the apparent activation energy (1.47 eV) was also much higher than Mo@NiCoOOH.)


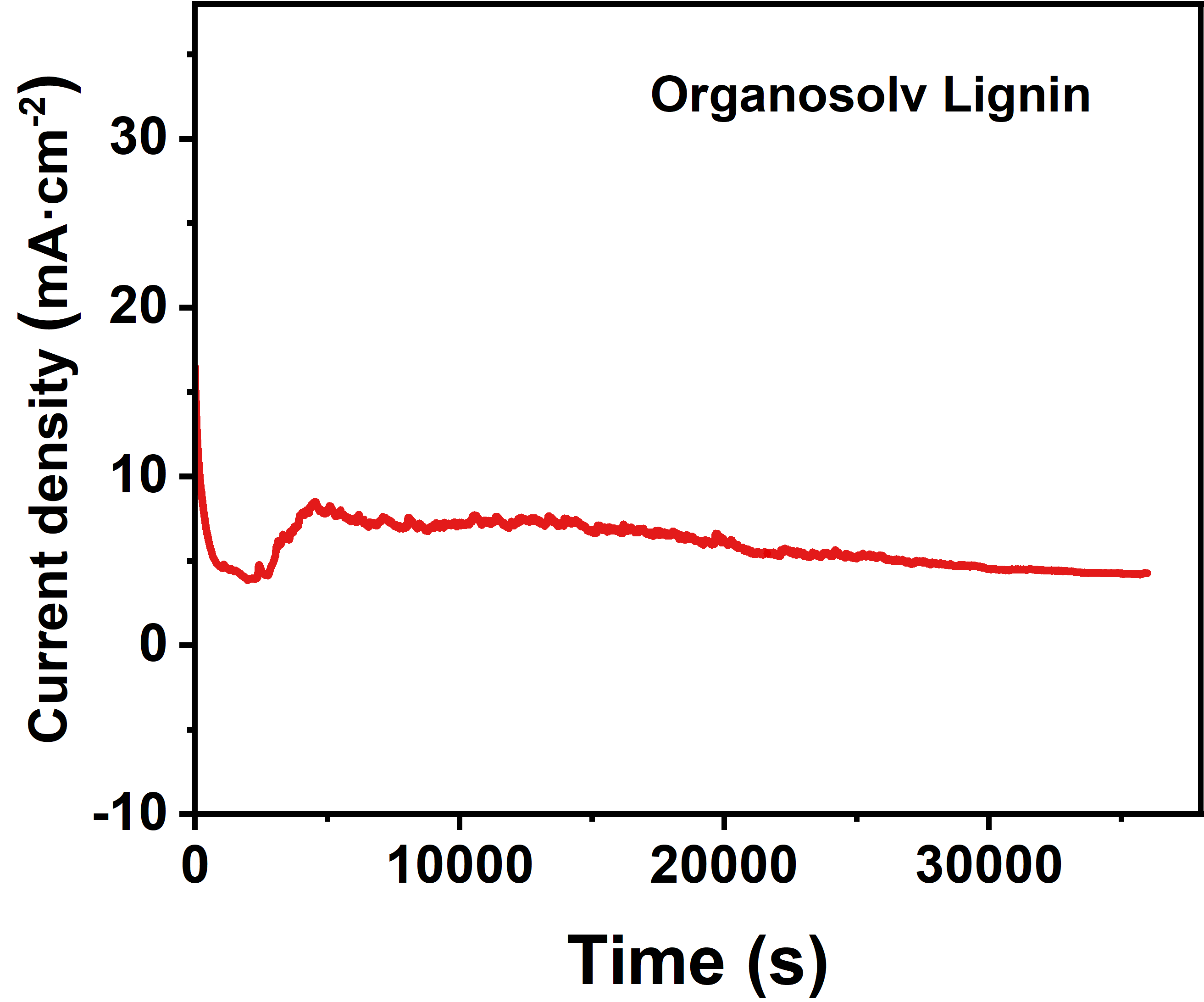


**Fig. S24.** The I-t curve of Mo@NiCoOOH for organosolv lignin under optimized reaction condition: OLs (50mg), *n*Bu_4_NOH (0.22 mmol), TBHP (1.2 mmol), 2-Me THF (5.0 mL), MeCN (5.0 mL), RT, *E* = 4.0 V vs Ag/AgCl, 10 h, under air.


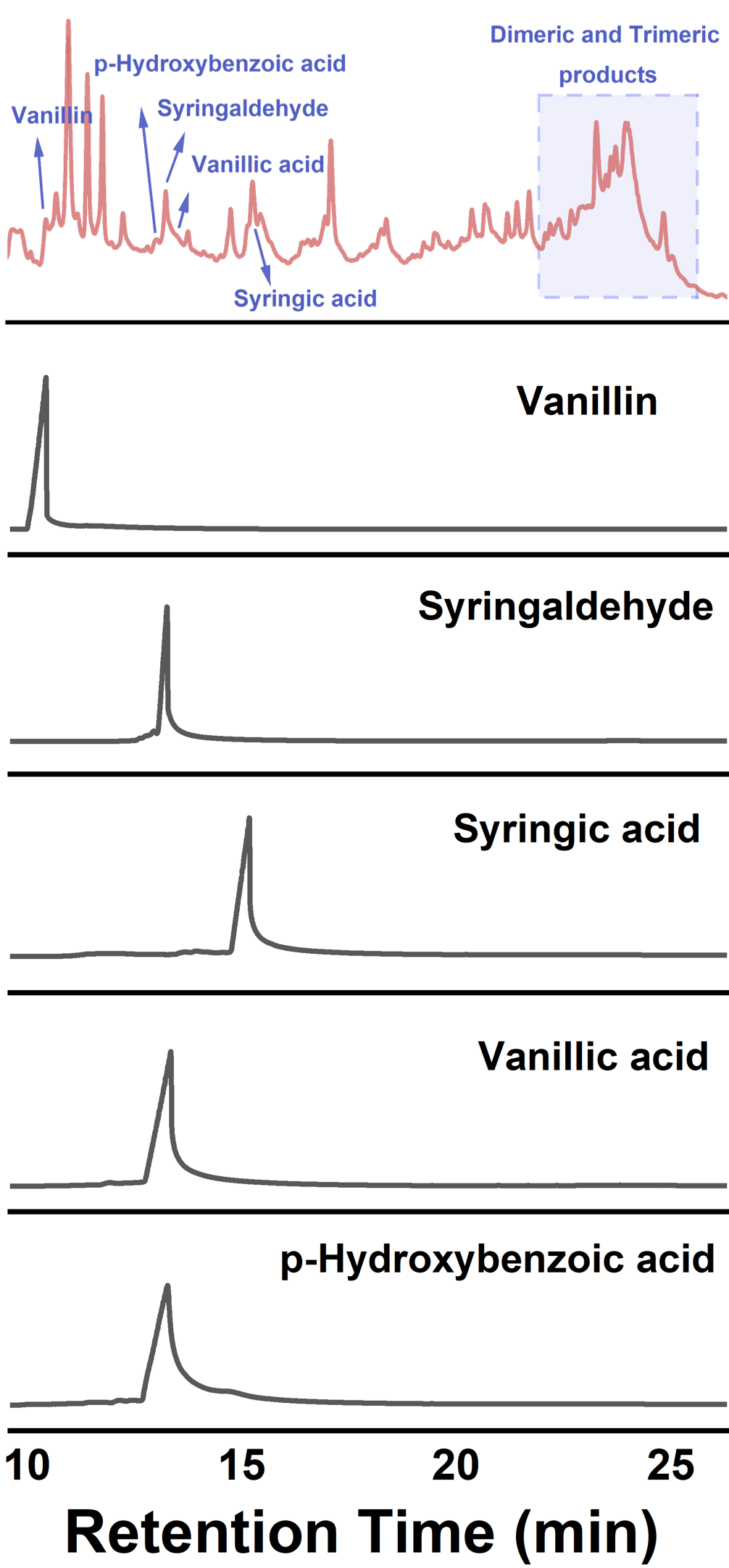


**Fig. S25.** GC-MS chromatograms for the reaction mixture after OL depolymerization match with the standard samples.


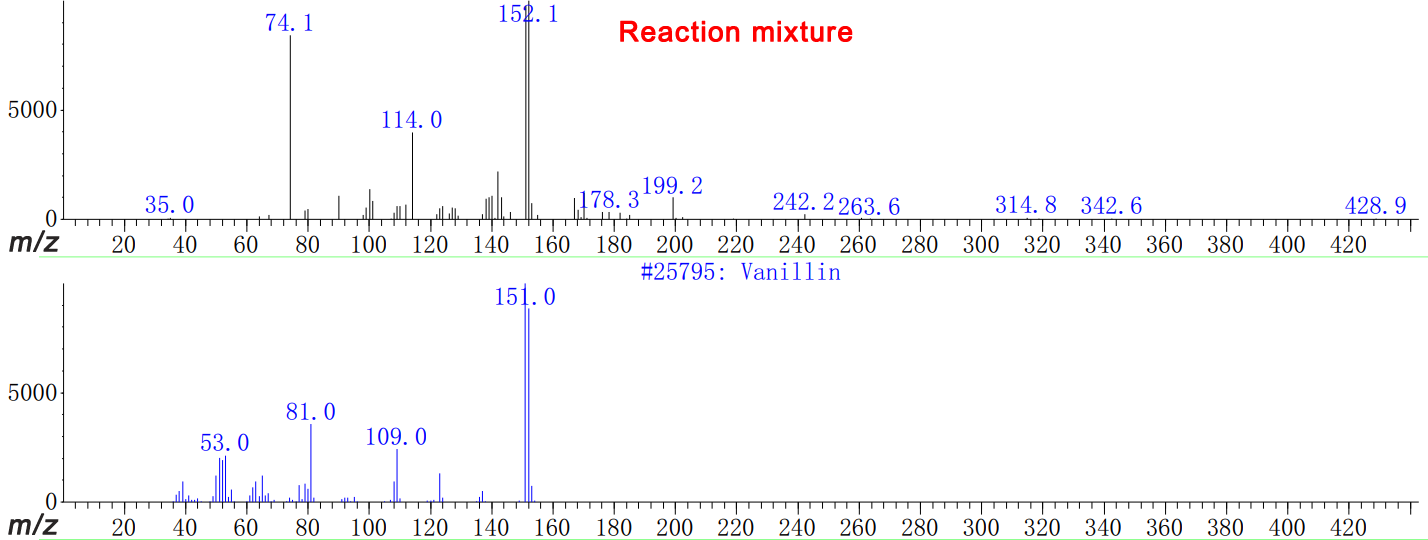


**Fig. S26.** Representative data used to establish the identity of lignin depolymerization products: Vanillin.


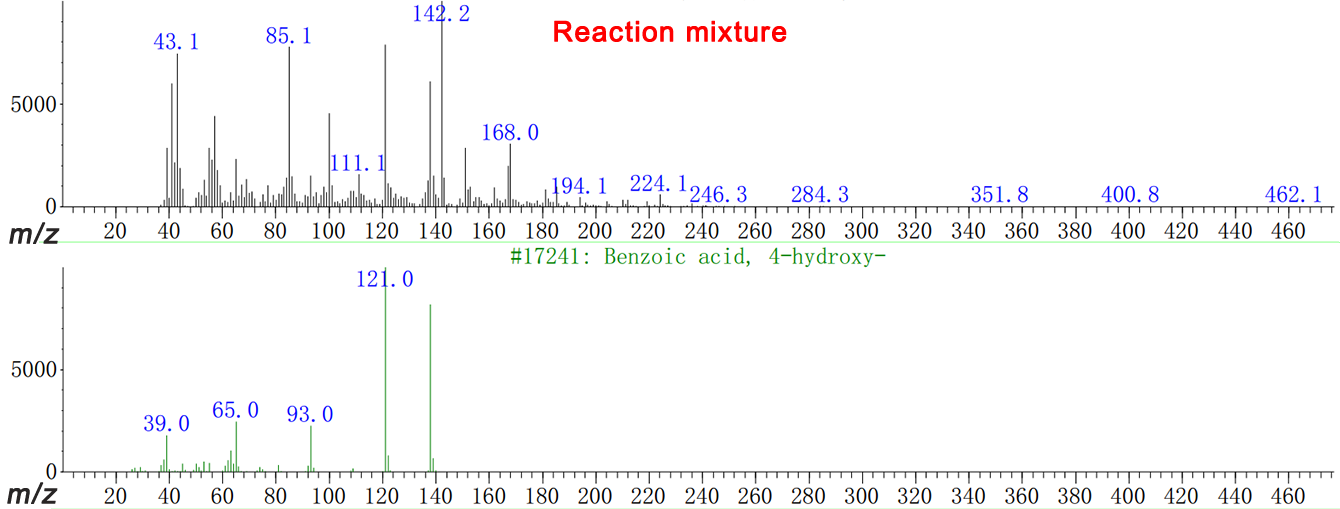


**Fig. S27.** Representative data used to establish the identity of lignin depolymerization products: *p*-Hydroxybenzoic acid.


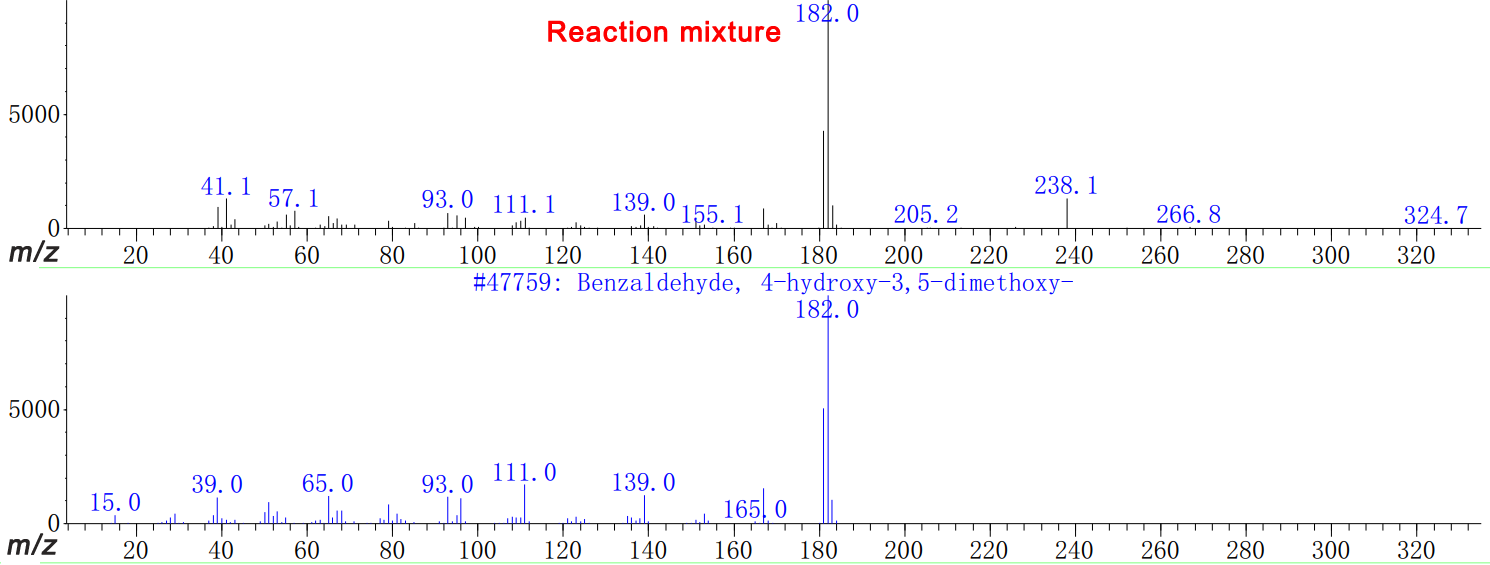


**Fig. S28.** Representative data used to establish the identity of lignin depolymerization products: Syringaldehyde.


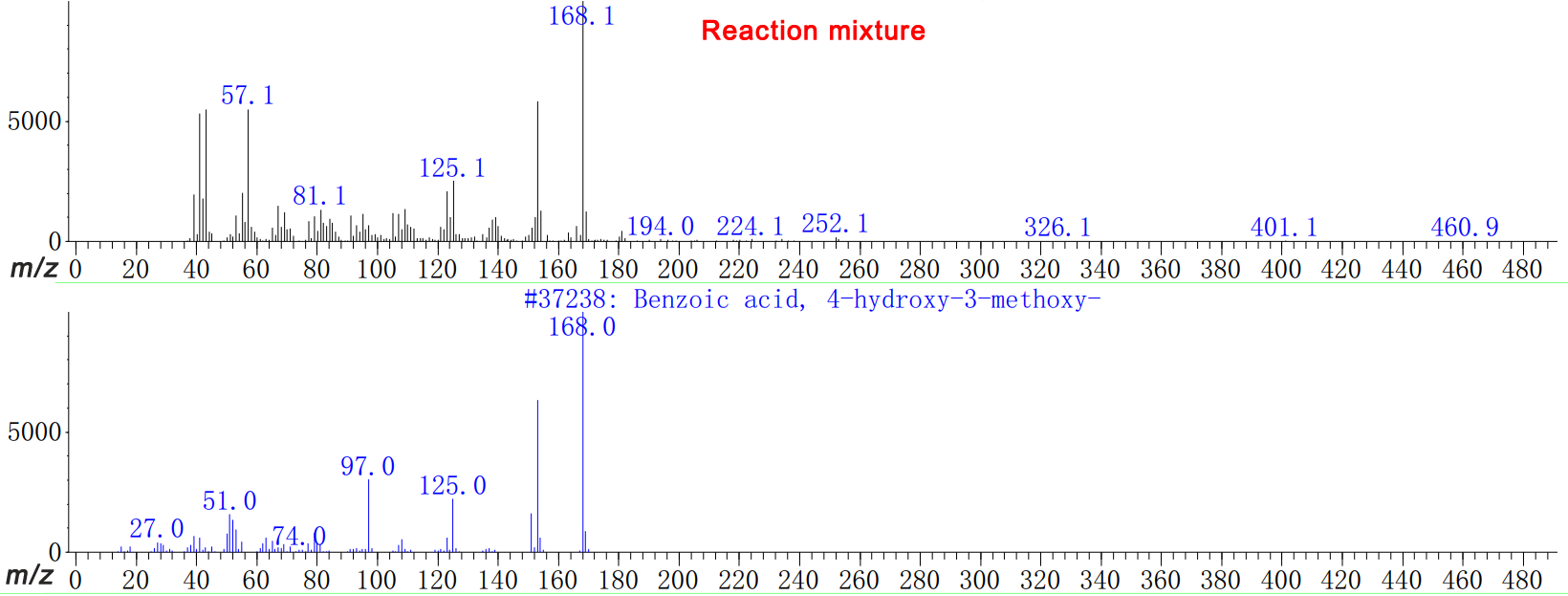


**Fig. S29.** Representative data used to establish the identity of lignin depolymerization products: Vanillic acid.


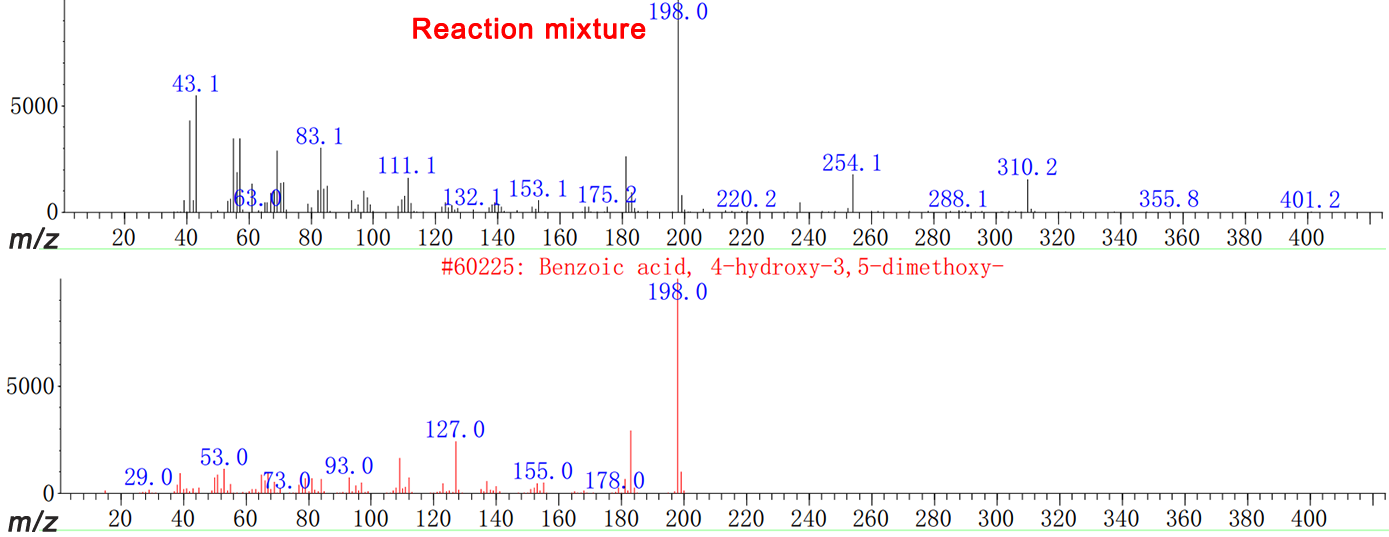


**Fig. S30.** Representative data used to establish the identity of lignin depolymerization products: Syringic acid.


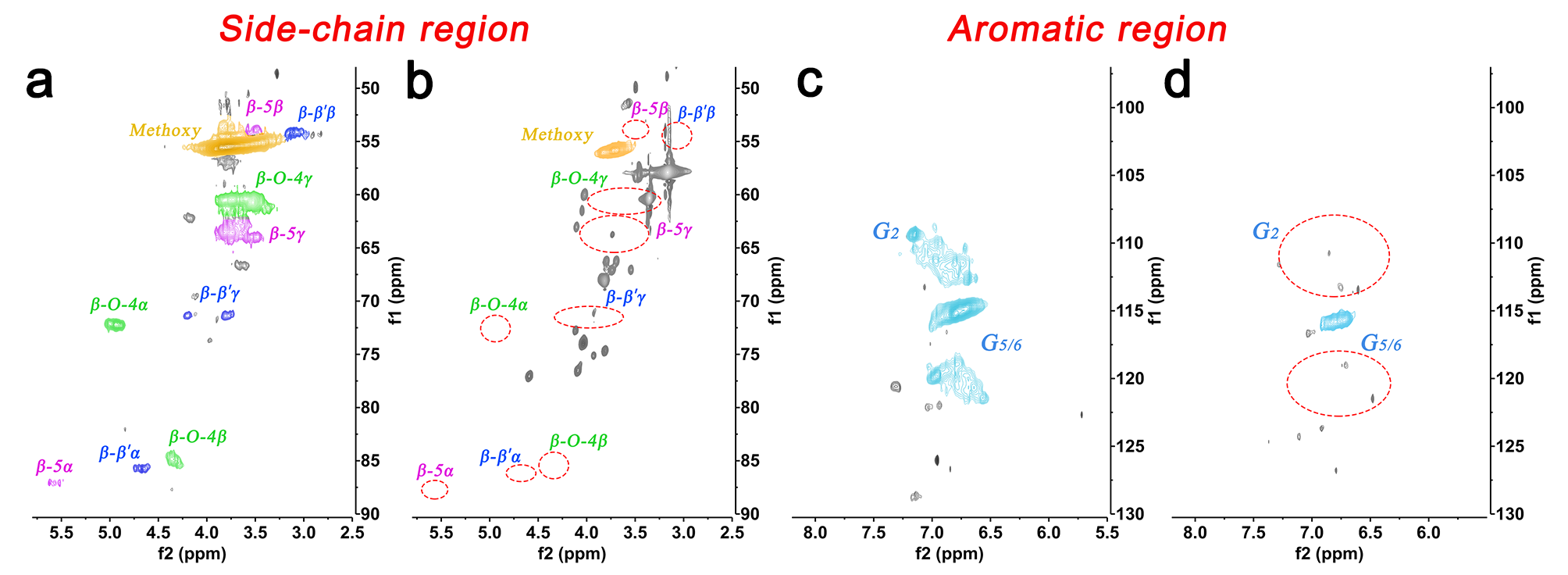


**Fig. S31.** 2D HSQC NMR spectra comparison of Kraft lignin (KL). (a) Before and (b) after the electrochemical reaction in side-chain regions. (c) Before and (d) after the electrochemical reaction in aromatic regions. Reaction conditions: KL (50mg), *n*Bu_4_NOH (0.22 mmol), TBHP (1.2 mmol), 2-Me THF (5.0 mL), MeCN (5.0 mL), RT, *E* = 4.0 V vs Ag/AgCl, 10 h, under air.


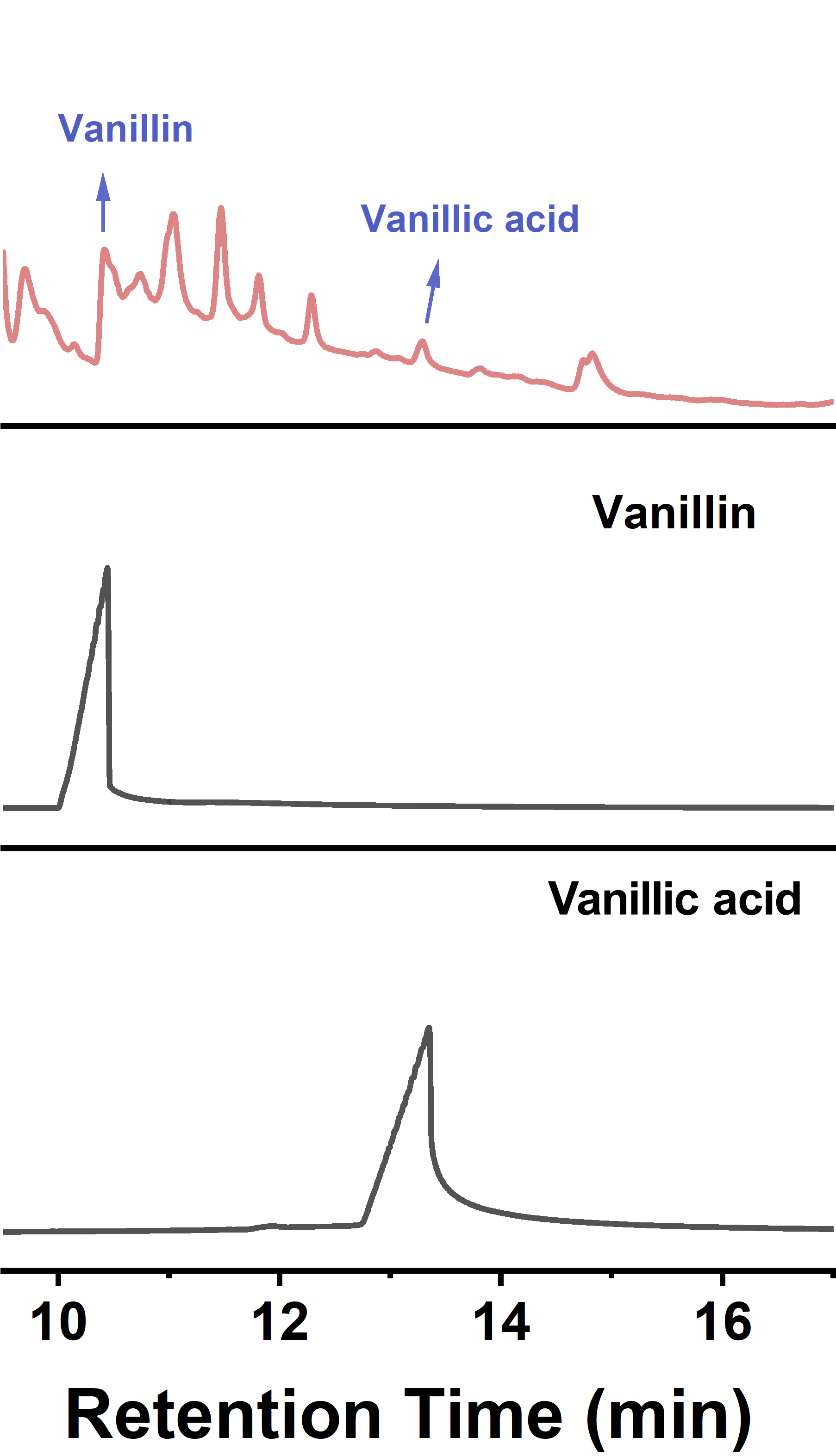


**Fig. S32.** GC-MS chromatograms for the reaction mixture after KL depolymerization match with the standard samples.

**Table S1.** The depolymerized performance of Mo@NiCoOOH for the oxidative cleavage of **1a** comparison in electro-, photo- and even thermos-catalytic approaches.

| **Catalyst** | **Substrate addition of 1a (mmol)** | **Reaction conditions** | **Con. of 1a (%)** | **C-C bond cleavage product yield (%)** | | | | **Ref.** |
| --- | --- | --- | --- | --- | --- | --- | --- | --- |
|  |  |  |  | **1b** | **1c** | **1d** | **1f** |  |
| Electrocatalytic approach | | | | | | | | |
| **Mo@NiCoOOH** | 0.2 | RT , air , ≈13mA, TBHP, 5h | 93.69 | 57.29 | 28.07 | - | 13 | **This work** |
| Pt_1_/N-CNTs | 0.1 | RT , air ,  20mA, TBHP, 5h | >99 | 81 | - | - | 56 | ^6^ |
| Pt | 0.2 | RT , air ,  20mA, TBHP, 3h | 93 | 67 | - | 27 | 27 | ^7^ |
| NF@Co_3_S_4_/(α,β)-NiS | 0.05 | RT , air , 1.414 V vs RHE, 8h | 93.6 | 0 | 83.8 | - | - | ^8^ |
| MnCoOOH/NF | 0.5 | RT , air, 1.5 V vs RHE, 8h | >99 | 0 |  | 84.1 | - | ^9^ |
| Photocatalytic approach | | | | | | | | |
| mpg-C_3_N_4_ | 0.05 | RT , 455 nm LED , 0.1 MPa O_2_, 10h | 96 | 51 | - | 21 | - | ^10^ |
| Ir-1 Catalyst | 0.1 | 50W Blue LED ,  N_2_ , collidine , PhSH, 12h | ＞99 | 90 | - | - | - | ^11^ |
| BiVO_4_ | 0.25 | AM 1.5 sunlight , 2.0 V vs Ag/AgCl, 20h | 30 | 12 | - | - | 5 | ^12^ |
| U-Nb_2_O_5_ HMS | 0.05 | 300W xenon , 0.1Mpa O_2_,18h | 94 | 48 | - | 25 | - | ^13^ |
| ILs [BMim][NTf_2_] | 0.02 | 60 °C,UV light, 100 mW cm^-2^, [PrHSO_3_Mim][OTf], 20min | 95.1 | 18 | - | - | 50.4 | ^14^ |
| Thermo-catalytic approach | | | | | | | | |
| LCN | 0.5 | 120 °C, TBHP, 24h | 91.8 | 0.2 | - | 45.3 | 18.3 | ^15^ |
| VO(acac)_2_ | 0.25 | 80 °C , 0.1Mpa, O_2_ ,8h | 89.2 | 4.3 | - | 28.7 | 44.2 | ^16^ |
| Cu(OAc)_2_-1,10-phenanthroline | 0.2 | 80 °C, 0.4 MPa O_2_ ,6h | ＞99 | - | - | 79 | - | ^17^ |
| MENU-MV-5 | 0.5 | 100 °C，0.4 MPa O_2_ ,12h | ＞99 | 6.5 | 90 | - | 60 | ^18^ |
| VB_12_@C-900 | 0.25mmol | 80 °C, K_2_CO_3_ , 0.1 MPa O_2_ ,24h | 96 | - | 73 | - | 96 | ^19^ |

**1a = 2-Phenoxy-1-phenylethanol**

**1b = Benzaldehyde**

**1c = Methyl Benzoate**

**1d = Benzoic Acid**

**1f = Phenol**

**Table S2.** The free energy (eV) during stepwise **1a** oxidation cleavage in route 1 by Mo@NiCoOOH.

| **Reaction step Pathway** | **Route 1** |
| --- | --- |
| 1a^*^ + BuO**·**^*^ | -2.230 |
| 1a’^*^ + BuOH^*^ | -2.187 |
| 1a’OO**·**^*^+ BuOH^*^ | -2.520 |
| 1a’OOH + BuO**·**^*^ | -1.199 |
| 1a’OH + BuO^*^+ [O] | -2.649 |
| 1b + 1e | -2.106 |

**Table S3.** The free energy (eV) during stepwise **1a** oxidation cleavage in Mo@NiOOH and Mo@NiCoOOH.

| **Reaction step Catalysts** | **Mo@NiOOH** | **Mo@NiCoOOH** |
| --- | --- | --- |
| 1a^*^+OH^*^ | -0.979 | -1.457 |
| TS1 | -0.423 | -1.001 |
| 1a**·**^*^+H_2_O^*^ | -1.893 | -1.242 |
| 1a**·**^*^+H_2_O (g) | -0.940 | -0.744 |
| 1a^*^+H_2_O (g) | 1.472 | 0.034 |
| 1a**·**-O*^t^*Bu | -0.936 | -2.374 |
| TS2 | -0.412 | -1.850 |
| 1b+1e+*^t^*BuH | -1.300 | -2.738 |

**Supplementary References**

1. Xie J, Zhang J, Li S, Grote F, Zhang X, Zhang H, Wang R, Lei Y, Pan B, Xie Y. Controllable disorder engineering in oxygen-incorporated MoS_2_ ultrathin nanosheets for efficient hydrogen evolution. *J. Am. Chem. Soc.* 2013; 135 (47): 17881-8.
2. He Z, Zhang J, Gong Z, Lei H, Zhou D, Zhang N, Mai W, Zhao S, Chen Y. Activating lattice oxygen in NiFe-based (oxy)hydroxide for water electrolysis. *Nat. Commun.* 2022; 13 (1): 2191.
3. Li S.-J, Guo W, Yuan B.-Q, Zhang D.-J, Feng Z.-Q, Du J.-M. Assembly of ultrathin NiOOH nanosheets on electrochemically pretreated glassy carbon electrode for electrocatalytic oxidation of glucose and methanol. *Sens. Actuators B* 2017; 240: 398-407.
4. Wu X, Fan X, Xie S, Lin J, Cheng J, Zhang Q, Chen L, Wang Y. Solar energy-driven lignin-first approach to full utilization of lignocellulosic biomass under mild conditions. *Nat. Catal.* 2018; 1 (10): 772-780.
5. Luo N, Wang M, Li H, Zhang J, Hou T, Chen H, Zhang X, Lu J, Wang F. Visible-Light-Driven Self-Hydrogen Transfer Hydrogenolysis of Lignin Models and Extracts into Phenolic Products. *ACS Catal.* 2017; 7 (7): 4571-4580.
6. Cui T, Ma L, Wang S, Ye C, Liang X, Zhang Z, Meng G, Zheng L, Hu H. S, Zhang J, Duan H, Wang D, Li Y. Atomically Dispersed Pt-N_3_C_1_ Sites Enabling Efficient and Selective Electrocatalytic C-C Bond Cleavage in Lignin Models under Ambient Conditions. *J. Am. Chem. Soc.* 2021; 143 (25): 9429-9439.
7. Ma L, Zhou H, Kong X, Li Z, Duan H. An Electrocatalytic Strategy for C–C Bond Cleavage in Lignin Model Compounds and Lignin under Ambient Conditions. *ACS Sustainable Che. Eng.* 2021; 9 (4): 1932-1940.
8. Wang N, Xue R, Yang N, Sun H, Zhang B, Ma Z, Ma Y, Zang L. Efficient oxidative cleavage of lignin C-C model compound using MOF-derived Cobalt/Nickel sulfide heterostructures. *J. Alloys Compd.* 2022; 929.
9. Zhou H, Li Z, Xu S. M, Lu L, Xu M, Ji K, Ge R, Yan Y, Ma L, Kong X, Zheng L, Duan H. Selectively Upgrading Lignin Derivatives to Carboxylates through Electrochemical Oxidative C(OH)-C Bond Cleavage by a Mn-Doped Cobalt Oxyhydroxide Catalyst. *Angew. Chem. Int. Ed.* 2021; 60 (16): 8976-8982.
10. Liu H, Li H, Lu J, Zeng S, Wang M, Luo N, Xu S, Wang F. Photocatalytic Cleavage of C–C Bond in Lignin Models under Visible Light on Mesoporous Graphitic Carbon Nitride through π–π Stacking Interaction. *ACS Catal.* 2018; 8 (6): 4761-4771.
11. Wang Y, Liu Y, He J, Zhang Y. Redox-neutral photocatalytic strategy for selective C-C bond cleavage of lignin and lignin models via PCET process. *Sci. Bull.* 2019; 64 (22): 1658-1666.
12. Li T, Mo J. Y, Weekes D. M, Dettelbach K. E, Jansonius R. P, Sammis G. M, Berlinguette C. P. Photoelectrochemical Decomposition of Lignin Model Compound on a BiVO_4_ Photoanode. *ChemSusChem* 2020; 13 (14): 3622-3626.
13. Chen H, Hong D, Wan K, Wang J, Niu B, Zhang Y, Long D. Urchin-like Nb_2_O_5_ hollow microspheres enabling efficient and selective photocatalytic C–C bond cleavage in lignin models under ambient conditions. *Chin. Chem. Lett.* 2022; 33 (9): 4357-4362.
14. Kang Y, Yao X, Yang Y, Xu J, Xin J, Zhou Q, Li M, Lu X, Zhang S. Metal-free and mild photo-thermal synergism in ionic liquids for lignin C_α_–C_β_ bond cleavage to provide aldehydes. *Green Chem.*2021; 23 (15): 5524-5534.
15. Gao Y, Zhang J, Chen X, Ma D, Yan N. A Metal-Free Carbon-Based Catalytic System for the Oxidation of Lignin Model Compounds and Lignin. *ChemPlusChem* 2014; 79 (6): 825-834.
16. Ma Y, Du Z, Liu J, Xia F, Xu J. Selective oxidative C–C bond cleavage of a lignin model compound in the presence of acetic acid with a vanadium catalyst. *Green Chem.* 2015; 17 (11): 4968-4973.
17. Wang M, Lu J, Li L, Li H, Liu H, Wang F. Oxidative C(OH)-C bond cleavage of secondary alcohols to acids over a copper catalyst with molecular oxygen as the oxidant. *J. Catal.* 2017; 348: 160-167.
18. Tian H.-R, Liu Y.-W, Zhang Z, Liu S.-M, Dang T.-Y, Li X.-H, Sun X.-W, Lu Y, Liu S.-X. A multicentre synergistic polyoxometalate-based metal–organic framework for one-step selective oxidative cleavage of β-O-4 lignin model compounds. *Green Chem.* 2020; 22 (1): 248-255.
19. Luo H, Wang L, Li G, Shang S, Lv Y, Niu J, Gao S. Nitrogen-Doped Carbon-Modified Cobalt-Nanoparticle-Catalyzed Oxidative Cleavage of Lignin β-O-4 Model Compounds under Mild Conditions. *ACS Sustain. Chem. Eng.* 2018; 6 (11): 14188-14196.
